# ATM controls meiotic DNA double-strand break formation and recombination and affects synaptonemal complex organization in plants

**DOI:** 10.1093/plcell/koab045

**Published:** 2021-02-05

**Authors:** Marie-Therese Kurzbauer, Michael Peter Janisiw, Luis F Paulin, Ignacio Prusén Mota, Konstantin Tomanov, Ondrej Krsicka, Arndt von Haeseler, Veit Schubert, Peter Schlögelhofer

**Affiliations:** 1 Department of Chromosome Biology, Max Perutz Labs, University of Vienna, Vienna BioCenter, Vienna, Austria; 2 Center for Integrative Bioinformatics Vienna (CIBIV), Max Perutz Labs, University of Vienna and Medical University of Vienna, Vienna BioCenter, Vienna, Austria; 3 Bioinformatics and Computational Biology, Faculty of Computer Science, University of Vienna, Vienna, Austria; 4 Leibniz Institute of Plant Genetics and Crop Plant Research (IPK) Gatersleben, 06466, Seeland, Germany

## Abstract

Meiosis is a specialized cell division that gives rise to genetically distinct gametic cells. Meiosis relies on the tightly controlled formation of DNA double-strand breaks (DSBs) and their repair via homologous recombination for correct chromosome segregation. Like all forms of DNA damage, meiotic DSBs are potentially harmful and their formation activates an elaborate response to inhibit excessive DNA break formation and ensure successful repair. Previous studies established the protein kinase ATM as a DSB sensor and meiotic regulator in several organisms. Here we show that Arabidopsis ATM acts at multiple steps during DSB formation and processing, as well as crossover (CO) formation and synaptonemal complex (SC) organization, all vital for the successful completion of meiosis. We developed a single-molecule approach to quantify meiotic breaks and determined that ATM is essential to limit the number of meiotic DSBs. Local and genome-wide recombination screens showed that ATM restricts the number of interference-insensitive COs, while super-resolution STED nanoscopy of meiotic chromosomes revealed that the kinase affects chromatin loop size and SC length and width. Our study extends our understanding of how ATM functions during plant meiosis and establishes it as an integral factor of the meiotic program.

## Teaser

Arabidopsis ATM acts at multiple steps during DSB formation and processing, as well as crossover formation and synaptonemal complex organization, all vital for the successful completion of meiosis.

## Introduction

Developmental transitions depend on the precise orchestration of intricate molecular processes and involve the tight regulation of protein functions and activities. One way to reversibly modulate these functions is via post-translational modifications (PTMs), such as phosphorylation (reviewed in Dissmeyer and Schnittger, 2011). Fundamental steps during cell division, as well as cellular stress responses—in particular, the DNA damage response (DDR)—are dependent on this rapid and short-lived PTM (Krylov et al., 2003). Central to the regulation of the DDR is the conserved kinase ATAXIA TELANGIECTASIA MUTATED (ATM; reviewed in Shiloh and Ziv, 2013; Blackford and Jackson, 2017). The protein name derives from a rare autosomal recessive human disorder, ataxia telangiectasia, which is characterized by dilated blood vessels (telangiectasia), progressive neurodegeneration triggering gait abnormalities (ataxia), premature aging, immunodeficiency, infertility, chromosomal instability, and a predisposition to cancer (reviewed in Blackford and Jackson, 2017). The underlying mutations were identified over 20 years ago and related to the *ATM* gene (Savitsky et al., 1995). ATM is a large protein of approximately 350 kD (Savitsky et al., 1995; Lavin et al., 2004) and is ordinarily present as a catalytically inactive dimer. In response to DNA damage and upon activation by the MRN (MRE11, RAD50, NBS1) complex, autophosphorylation leads to monomerization of ATM (reviewed in Paull, 2015). Once activated, monomeric ATM phosphorylates a large number of target proteins involved in cell-cycle checkpoints, DNA repair, and several other cellular processes (reviewed in Paull, 2015), preferably at Ser/Gln or Thr/Gln sites (Smolka et al., 2007; Bennetzen et al., 2010; Roitinger et al., 2015).

**Figure:**
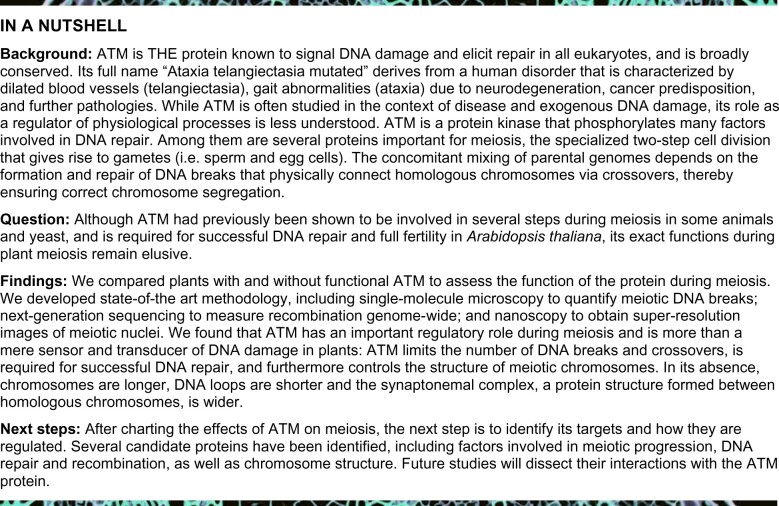


Studies in several organisms have furthermore revealed that ATM is a major player in meiosis, as it affects fertility, meiotic DNA double-strand break (DSB) formation, spatial DSB patterning, and DNA repair (Garcia et al., 2003; Joyce et al., 2011; Lange et al., 2011; Zhang et al., 2011; Carballo et al., 2013; Anderson et al., 2015; Garcia et al., 2015; Mohibullah and Keeney, 2017; Fowler et al., 2018; Lukaszewicz et al., 2018; Tian and Loidl, 2018; Li and Yanowitz, 2019). Meiosis is a specialized, two-step cell division that gives rise to genetically distinct gametic cells. During the first meiotic division, homologous chromosomes pair, recombine, and are subsequently separated to opposite poles of the cell. Without intervening DNA replication, sister chromatids are then separated during the ensuing second division. Meiosis is completed by the formation of four genetically different haploid precursor cells that further develop into gametic cells. The timely and spatially coordinated formation of DSBs and their repair is essential for pairing and recombination of homologous chromosomes, and thereby for successful meiotic division. Various members of the DSB-forming complex have been identified, including the conserved topoisomerase-related SPOrulation 11 (SPO11) protein, which acts as the catalytically active factor in the complex (reviewed in Keeney, 2001; Edlinger and Schlogelhofer, 2011; Robert et al., 2016). After DSB formation, SPO11 is released from the break site by the MRE11/RAD50/Xrs2-NBS1 (MRX/N) complex in conjunction with COM1 (Completion of Meiotic Recombination, also named Sporulation in the Absence of spo Eleven [SAE2]) and remains covalently bound to an oligonucleotide (Neale et al., 2005; Uanschou et al., 2007; Milman et al., 2009). The DNA ends are resected, stabilized, and loaded with the highly conserved recombinases RAD51 and DMC1 (Disruption of Meiotic Control1), which facilitate strand invasion into homologous sequences (reviewed in Crickard and Greene, 2018). Depending on the template and the resolution of the intermediate structures, DNA repair results in the formation of either crossover (CO) or noncrossover (NCO) molecules (reviewed in Hunter, 2007; Broderick et al., 2010; Osman et al., 2011). In a variety of organisms, including Arabidopsis (*Arabidopsis thaliana*), a surplus of meiotic DSBs forms but only a fraction is repaired by connecting maternal and paternal chromosome arms to form a CO (Buhler et al., 2007; Sanchez-Moran et al., 2007; Vignard et al., 2007). COs contribute to the exchange of parental genetic material and physically link homologous chromosomes, thereby enabling their correct segregation during meiosis I. Two classes of COs exist in most organisms: class I or interfering COs inhibit the formation of further COs nearby, whereas class II COs are non-interfering and form independently of other recombination events (reviewed in Wang et al., 2015). The remaining breaks are repaired via intersister recombination, or are repaired to yield NCO products, possibly utilizing a synthesis-dependent strand-annealing (SDSA) pathway (Higgins et al., 2004; Chen et al., 2008; Mancera et al., 2008).

Like all forms of DNA damage, meiotic DSBs are potentially harmful and their formation and repair are tightly controlled. They are non-randomly distributed along chromosomes and form preferentially in specific regions of the genome termed DSB hotspots (Kauppi et al., 2004; Pan et al., 2011; Tischfield and Keeney, 2012; Lange et al., 2016). In plants, hotspots are typically located in nucleosome-depleted regions and gene promoters containing AT-rich DNA sequences (reviewed in Tock and Henderson, 2018). In every nucleus, a sufficient number of DSBs needs to be introduced to enable the formation of at least one CO per chromosome and thus ensure the correct segregation of homologous chromosomes during anaphase I. It is imperative that the number, timing, and location of DSB formation are tightly regulated (reviewed in Keeney et al., 2014). ATM and the related kinase ATR (ataxia telangiectasia mutated and Rad3-related) have been implicated in the negative regulation of DSB formation, locally and in trans (on the sister chromatid) and in implementing DSB interference (Joyce et al., 2011; Lange et al., 2011; Zhang et al., 2011; Carballo et al., 2013; Garcia et al., 2015; Mohibullah and Keeney, 2017; Fowler et al., 2018; Li and Yanowitz, 2019).

The regulatory functions of ATM during meiosis are not limited to DSB formation but extend to DSB processing, DNA repair template choice, and CO formation. Studies in budding yeast (*Saccharomyces cerevisiae*) showed that non-interfering COs specifically increase in number in the absence of TELomere maintenance 1 (Tel1, the yeast homolog of ATM). Numbers of MutL homolog 1 (MLH1) foci on meiotic chromosome spreads marking class I CO sites are increased and their interference perturbed on *Atm* mutant mice autosomes. CO numbers are generally diminished in nematode (*Caenorhabditis elegans*) *atm-1* mutants and the obligate XY CO is lost in mouse (*Mus musculus*) spermatocytes lacking ATM function (Barchi et al., 2008; Anderson et al., 2015; Mimitou et al., 2017; Li and Yanowitz, 2019).

The Arabidopsis *ATM* homolog shares 58% identity and 67% similarity with the human gene and the encoded protein is 3,856 amino acids, with a predicted molecular weight of 440 kD (Garcia et al., 2000). The Arabidopsis ATM protein has been shown to play a role in DNA damage sensing, DNA repair, and fertility and has only recently been implicated in regulating DSB or CO formation (Garcia et al., 2003; Culligan et al., 2004; Culligan et al., 2006; Culligan and Britt, 2008; Kurzbauer et al., 2018; Yao et al., 2020; Zhang et al., 2020). Analysis of spreads of pollen mother cells (PMCs) revealed extensive chromosome fragmentation during meiosis. These results confirmed comparable observations initially made in mammals and mammalian cell lines and led to the assumption that ATM responds specifically to DSBs in somatic and meiotic cells in a variety of organisms, including plants (Xu and Baltimore, 1996; Rotman and Shiloh, 1998; Garcia et al., 2003).

Our analysis extends the understanding of the role of ATM in regulating key events of Arabidopsis meiosis. We discovered that ATM is required at multiple steps during meiosis; it has an evolutionarily conserved role in limiting the number of meiotic DSBs, is required for successful DNA repair and, in its absence, additional MMS and UV Sensitive 81 (MUS81)-dependent COs form that perturb interference patterns. We furthermore showed that synaptonemal complex (SC) length and width, as well as chromatin loop size, are regulated by ATM. Taken together, we demonstrate that the kinase ATM has an essential regulatory role during meiosis beyond that of a mere sensor and transducer of DNA damage in plants.

## Results

### ATM is important for successful meiotic DNA repair and full fertility

**Figure 1:**
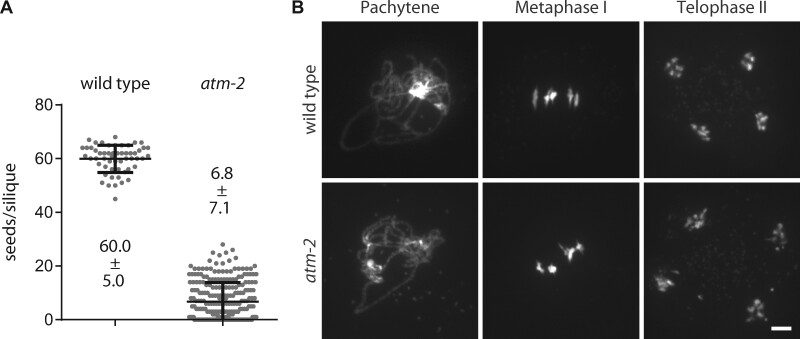
ATM is required for normal meiotic progression and full fertility. A, Whereas Columbia-0 wild-type plants produce 60.0 (±5.0) seeds per silique on average, *atm-2* mutant plants display reduced fertility with only 6.8 (±7.1) seeds per silique (*n* = 55 wild-type and 267 *atm-2* siliques). Error bars represent SDs. B, Meiosis appears unperturbed up to the pachytene/diplotene stage. Metaphase I cells sometimes display entanglements and chromosome fragments become visible in later stages, indicative of DNA repair defects. Bar: 5 µm.

We started our analysis by confirming the previously published observation that *atm-2* mutant plants bear siliques of variable length containing 6.8 ± 7.1 seeds on average, whereas wild-type plants produced 60.0 ± 5.0 seeds per silique (*n* = 55, wild-type; *n* = 267, *atm-2*; Figure 1A, Supplemental Figure S1A). We obtained similar numbers with a second mutant line, *atm-1* (Supplemental Figure S1B). Spread chromatin of PMCs appeared normal during meiotic progression up to the diplotene stage, but chromosome fragmentation did become visible at anaphase/telophase I in the mutants (Figure 1B, Supplemental Figure S2A; Garcia et al., 2003). Fragmentation was dependent on meiotic DSB formation, as *atm-2 spo11-2-3* double mutants formed intact univalents (Supplemental Figure S2B), corroborating earlier studies using different mutant alleles (Culligan and Britt, 2008). In addition, we observed chromosome bridges in 8% of nuclei between the anaphase I and tetrad stages (*n* = 53; Supplemental Figure S2C) suggesting that alternative repair pathways like non-homologous end joining may be at play in the absence of ATM (Friesner and Britt, 2003).

**Figure 2:**
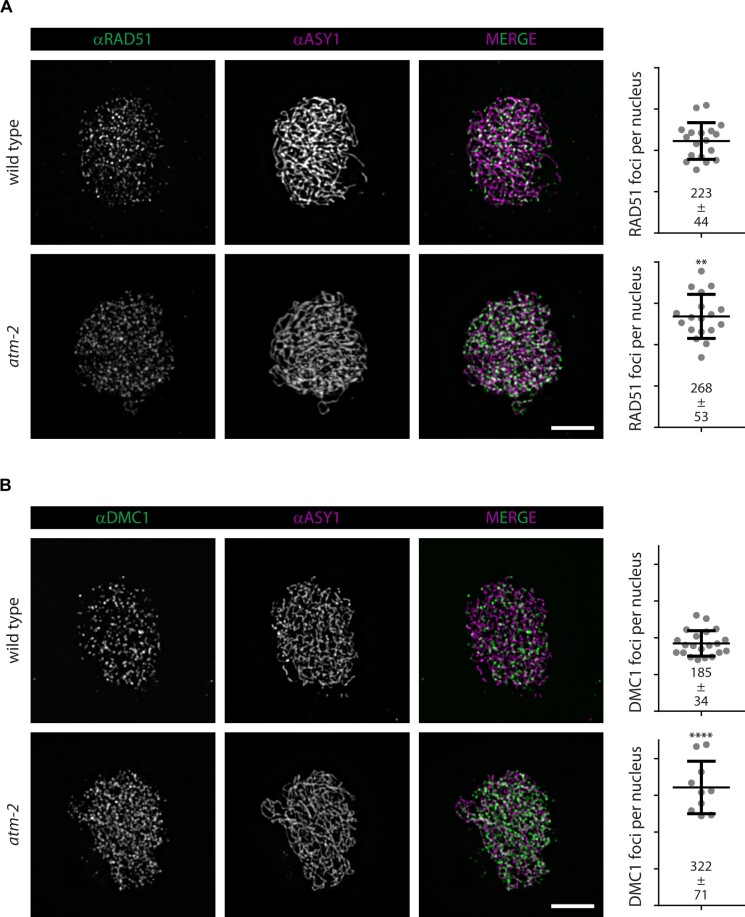
Recombinase foci numbers are elevated in *atm-2*. A, Wild-type meiocytes display 223 ± 44 RAD51 foci (*n* = 18 nuclei), whereas 268 ± 53 RAD51 foci are observed in *atm-2* mutant nuclei (*n* = 18 nuclei), representing a significant (*P* = 0.0086) increase. B, DMC1 foci are drastically increased from 185 ± 34 foci in the wild type (*n* = 21 nuclei) to 322 ± 71 foci in *atm-2* mutant nuclei (*n* = 10 nuclei; *P* < 0.0001). Size bars: 5µm. Error bars represent SDs.

To understand which aspects of meiotic DSB repair depend on ATM, we performed immunohistochemical staining for the recombinases RAD51 and DMC1. DNA repair is initiated even in the absence of *atm-2* and the recombinases are loaded onto chromatin—most likely due to the related kinase ATR (Barchi et al., 2008; Culligan and Britt, 2008). In *atm-2* zygotene nuclei, we observed significantly more RAD51 foci (268 ± 53 foci per nucleus, *n* = 18 nuclei) compared to the wild type (223 ± 44, *n* = 18 nuclei; *P* = 0.0117; Figure 2A). We obtained similar numbers of foci in a second mutant line, *atm-1* (279 ± 35 foci per nucleus, *n* = 14 nuclei), and the corresponding Wassilewskija wild type (Ws; 223 ± 23, *n* = 13 nuclei; *P* < 0.0001; Supplemental Figure S3). This difference was even more pronounced when we analyzed DMC1 foci, with only 185 ± 34 foci in wild-type PMCs (*n* = 21 nuclei) and 322 ± 71 in *atm-2* (*n* = 10 nuclei; *P* < 0.0001; Figure 2B). The increase in recombinase foci numbers may reflect increased DSB formation or a slowed turnover of recombination intermediates. Since the two recombinases RAD51 and DMC1 are differentially affected, a combination of increased DSB numbers and altered repair dynamics in the absence of ATM is possible.

### ATM is a negative regulator of DSB formation

**Figure 3:**
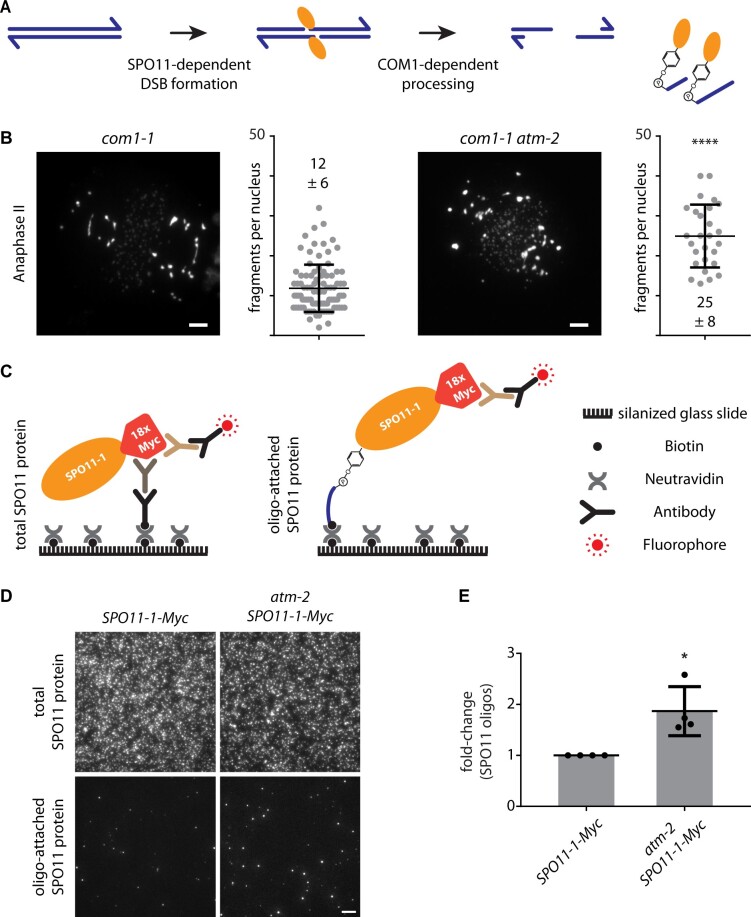
ATM limits DSB numbers. A, DSBs are formed by a protein complex with SPO11 (orange oval) being the catalytically active subunit. Incision, followed by COM1-dependent nucleolytic processing, releases SPO11 together with a covalently attached short oligonucleotide. B, DNA fragmentation as an indirect readout for DSB levels in DSB-processing deficient *com1-1* mutant plants. The number of fragments significantly increased from 12 in *com1-1* (±6; *n* = 94 nuclei) to 25 (±8; *n* = 27 nuclei; *P* < 0.0001) in *com1-1 atm-2*. Bar: 5 µm. C, Functionalized slides were used to immobilize total or oligo-attached SPO11 protein. D, SIM-TIRF microscopy was used to quantify single molecules of total SPO11-1-Myc protein and oligo-attached SPO11-1-Myc in wild-type and *atm-2* meiocytes. Bar: 5 µm. E, After normalization to total SPO11-1-Myc protein levels, a 1.87-fold increase of oligo-attached SPO11 was found in the *atm-2* mutant (*P* = 0.0286; four independent experiments). Error bars represent SDs.

Motivated by results obtained in other organisms that place ATM/Tel1 in a negative feedback loop controlling DSB numbers (Joyce et al., 2011; Lange et al., 2011; Zhang et al., 2011; Carballo et al., 2013; Garcia et al., 2015; Mohibullah and Keeney, 2017; Li and Yanowitz, 2019) and the observed increase in the numbers of recombinase foci in *atm-2* mutant plants, we analyzed DSB levels in ATM-deficient nuclei. We reasoned that the number of chromosome fragments visible in chromatin spreads of DNA repair mutants would serve as an indirect readout for DSB levels. We took advantage of the *com1-1* mutant background, where meiotic DSBs are formed but cannot be processed (Figure 3A; Uanschou et al., 2007). We scored fragments in metaphase II/anaphase II PMCs of *com1-1* mutants and counted 12 ± 6 (*n* = 94 nuclei) fragments per nucleus in the *com1-1* mutant line. The number of chromosome fragments doubled to 25 ± 8 (*n* = 27 nuclei; *P* < 0.0001) in the *com1-1 atm-2* background, highlighting the involvement of ATM in limiting DSB numbers in plants (Figure 3B). To validate our experimental setup, we exposed *com1-1* plants to ionizing radiation (100 Gy) to induce additional DSBs and observed a significant increase to 15 ± 4.6 fragments per nucleus (*n* = 55; *P* < 0.0001; observation 25 h after treatment; Supplemental Figure S4A).

To corroborate this finding, we developed an independent experimental approach based on single-molecule microscopy (Jain et al., 2012). This newly established technique accounts for limited starting material available (as in Arabidopsis flower buds) and allows for assessment of DSB formation in a more direct manner by quantitatively analyzing SPO11–oligonucleotide complexes—the byproducts of meiotic DSB formation (Figure 3A; Lange et al., 2011). The experimental setup relies on the immobilization and subsequent detection of total and oligonucleotide-bound SPO11 molecules from meiotic protein extracts. After activation and silanization, glass coverslips were biotinylated and coated with NeutrAvidin to serve as immobilization platform for SPO11 proteins via biotinylated antibodies, or SPO11 protein-bound oligonucleotides via terminal deoxynucleotidyl transferase dUTP nick end labeling (TUNEL)-based biotin-labeling of oligonucleotides (Figure 3C). We then detected immobilized proteins carrying multiple Myc tags by single-molecule total internal reflection fluorescence (SIM-TIRF) microscopy and quantified the number of fluorescent foci in the wild-type and mutant backgrounds (Figure 3D).

The system was set up using *S. cerevisiae* and fission yeast (*Schizosaccharomyces pombe*) and then optimized for the small amounts of starting material retrievable from Arabidopsis meiotic flower buds. We prepared meiotic protein extracts from *S. cerevisiae* strains expressing Spo11 with an 18× Myc tag (Prieler et al., 2005) in the presence and absence of functional Tel1/ATM. We then immunoprecipitated Spo11 via the Myc tag and split the precipitate into two fractions. With the first fraction, we determined the abundance of total Spo11 protein by direct immobilization and detection. Following biotinylation of the 3′ termini of Spo11-attached oligonucleotides from the second fraction, we immobilized the oligonucleotides and detected the covalently attached Spo11 to determine the amount of Spo11 proteins that had taken part in a cleavage reaction (i.e., DSB formation; Supplemental Figure S4B; refer to Materials and Methods section for further details). We quantified signals from Spo11-Myc18-oligonucleotides and total Spo11-Myc18 proteins from independent images and normalized the results to lysate volume. We observed a 2.11-fold increase (±0.91, *P* = 0.0079, five biological replicates) of Spo11-Myc18-oligos in the *tel1Δ* mutant relative to wild type (Supplemental Table S1; Supplemental Figure S4C). This result was in line with previous studies using a different approach to quantify Spo11-oligos in *S. cerevisiae* where a 2.2-fold increase was found in *tel1Δ* strains (Mohibullah and Keeney, 2017) and validated our experimental approach. We similarly processed and evaluated protein extracts from meiotic *S. pombe* cultures expressing 13× Myc-tagged Rec12 (Spo11; Supplemental Figure S4D). Experiments were performed in wild-type and *rec10-155* mutant backgrounds (Wells et al., 2006). Rec10 is part of the *S. pombe* linear elements and is required to stimulate Rec12-mediated DSB formation. In *rec10-155* hypomorphic mutants, meiotic DSB formation and recombination were reduced (Mallela et al., 2011), as reflected by the steep decrease of oligo-attached Rec12 seen with SIM-TIRF microscopy (Supplemental Figure S4E; Supplemental Table S1).

To perform the same analysis in Arabidopsis, we generated a plant line expressing a functional 18× Myc-tagged SPO11-1 protein, which we then crossed to plants heterozygous for the *atm-2* insertional allele. Plants homozygous for the *SPO11-1-Myc18* transgene and for either the wild-type *ATM* allele (*SPO11-1-Myc18*) or the mutant allele (*atm-2 SPO11-1-Myc18*) were analyzed in the subsequent generation after selfing. Protein extracts were prepared, split, immobilized, detected, and signals evaluated as described above (Figure 3D). We found a 1.87-fold (±0.48, *P* = 0.0286, four biological replicates) increase of SPO11-1-Myc18-oligos in *atm-2* relative to the wild type (Supplemental Table S1, Figure 3E) and thus confirmed that DSB formation is indeed negatively regulated by ATM in Arabidopsis.

### ATM limits meiotic CO formation

**Figure 4:**
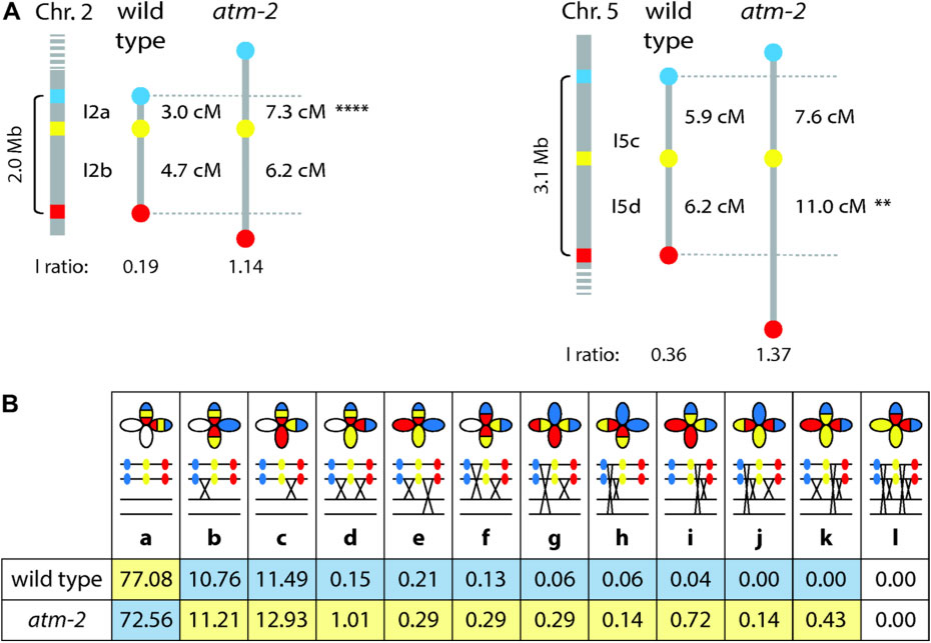
ATM limits recombination. A, CO frequencies in two neighboring intervals on chromosomes 2 and 5 were analyzed. Recombination significantly increased in two of the four intervals in the *atm-2* mutant and interference ratios dramatically increased (**P ≤ 0.01, ****P ≤ 0.0001; see text for details). I2a: FTL1506, eCFP, and FTL1524, eYFP; 0.5 Mb. I2b: FTL1524 and FTL965, DsRed2; 1.5 Mb. I5c: FTL 1963, eCFP, and FTL 1143, eYFP; 1.4 Mb. I5d: FTL1143 and FTL 2450, DsRed2; 1.7 Mb. B, Whereas non-recombinant tetrads appear less frequently in *atm-2* mutant plants, complex recombination events are specifically increased. Lower levels are shaded in blue, higher levels in yellow. Data for I5cd.

Prompted by the increase in DSB numbers in the *atm-2* mutant, we explored whether this would affect recombination rates. We used the fluorescent-tagged line (FTL) system (Francis et al., 2007; Berchowitz and Copenhaver, 2008) to analyze meiotic recombination rates and CO interference. This visual assay relies on the segregation of genetically linked transgenes that express fluorescent proteins in pollen. Mutation of the *QUARTET1* gene enables tetrad analysis, since all four pollen grains originating from a single PMC remain physically attached (Preuss et al., 1994; Francis et al., 2006). Assaying marker distributions in pollen tetrads allows for the calculation of genetic distances within each interval, and CO interference between adjacent intervals (Francis et al., 2007; Berchowitz and Copenhaver, 2008). We determined CO frequencies in two neighboring intervals—constituted by three transgenes—on chromosome 5 in wild-type and *atm-2* mutant pollen (Figure 4). Interval I5c (1.4 Mb) spans 5.9 centimorgans (cM) and I5d (1.7 Mb) 6.2 cM in wild-type plants (*n* = 4,682 pollen tetrads from five plants; Kurzbauer et al., 2018). We observed an increase in recombination frequency that did not reach statistical significance for interval I5c (7.6 cM; *P* = 0.0669) in the *atm-2* mutant. However, recombination did significantly increase to 11 cM in the I5d interval in the mutant (*n* = 696 pollen tetrads from eight plants; *P* = 0.0040). We obtained similar results for two additional intervals on chromosome 2 (I2a and I2b). We detected no significant difference in recombination frequency for interval I2b (1.5 Mb; 4.7 to 6.2 cM; *P* = 0.0696) in *atm-2* mutant plants, but recombination did significantly increase in the I2a interval (0.5Mb), from 3 cM in wild-type to 7.3 cM in *atm-2* mutant plants (*P* < 0.0001; wild type: *n* = 4,542 pollen tetrads from 5 plants; *atm-2*: *n* = 535 pollen tetrads from 16 plants; Figure 4A and B). We then determined interference ratios by calculating the ratio between the map distance of one of the intervals without and with a CO in the adjacent interval (Malkova et al., 2004). While interference ratios were normal in all analyzed regions in wild-type plants, they dramatically increased in the *atm-2* mutant (interference ratios I2ab: wild type: 0.19; *atm-2*: 1.14; I5cd wild type: 0.36; *atm-2*: 1.37; Figure 4A). This result may be explained by a perturbation of the interfering class I CO pathway or an increase in non-interfering class II COs. The ratios in *atm-2* reached values above one, indicating that COs are not spaced with a certain distance but rather tend to cluster, as has been suggested previously for budding and fission yeast *tel1* mutants (Zhang et al., 2011; Fowler et al., 2018). We confirmed this hypothesis by evaluating the nature of the recombination events. Non-recombinant tetrads appeared less frequently in the *atm-2* mutant than in wild type and all types of recombination events (single, double, and triple COs) increased. We only observed especially complex recombination patterns, like those involving three COs in the evaluated intervals, in mutant but never in wild-type pollen tetrads (Figure 4B).

**Figure 5:**
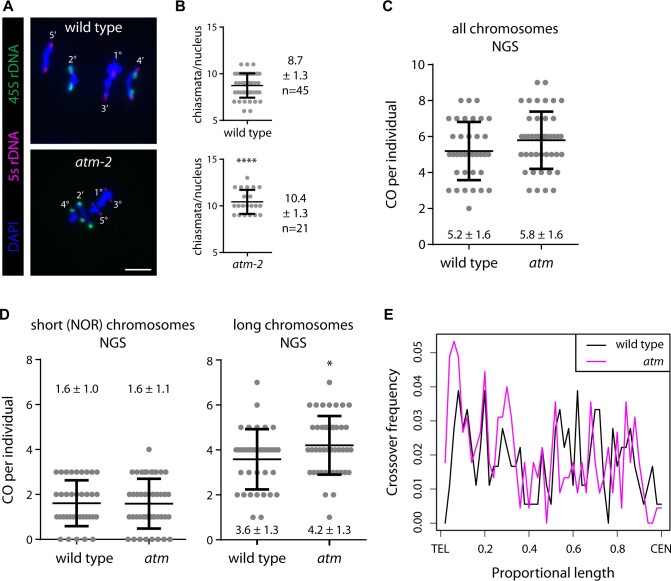
Chiasma and CO frequency increase in the absence of functional ATM. A, Chromosome spreads of wild-type (Col-0) and *atm-2* metaphase I nuclei. FISH probes against 45S (green) and 5S (magenta) rDNA were used to identify chromosomes. Chromosome identities and bivalent shapes are indicated (rod bivalent: ‘; ring bivalent: °). Bar: 5 µm. B, Number of chiasmata per nucleus, as determined by bivalent shape. C, On average 5.2 ± 1.6 COs in wild type and 5.8 ± 1.6 COs in *atm* mutant plants were detected by NGS. D, CO numbers do not significantly differ between wild-type and *atm* mutant plants when comparing short (NOR-bearing) chromosomes 2 and 4 (1.6 ± 1 and 1.6 ± 1.1; *P* = 0.9332) but are significantly increased on long chromosomes 1, 3, and 5 of *atm* mutant plants (3.6 ± 1.3 and 4.2 ± 1.3; *P* = 0.0395). E, CO frequency displayed in relation to the proportional length of all chromosome arms. The patterns for wild-type and mutant plants are very similar, with an increase of CO frequency closer to the telomeres in *atm* (*P ≤ 0.05; ****P ≤ 0.0001). Error bars represent SDs.

After assessing recombination rates in precisely defined genomic regions, we analyzed them genome-wide. We first evaluated meiotic chromosomes by 4,6-diamindine-2-phenylindole (DAPI) staining and fluorescent in situ hybridization (FISH) of metaphase I spreads to count chiasmata on individual bivalents (Sanchez Moran et al., 2001; Armstrong, 2013). Whereas wild-type nuclei displayed on average 8.7 (±1.3; *n* = 45 nuclei) chiasmata per nucleus, *atm-2* mutant nuclei showed a significant increase to 10.4 (±1.3; *n* = 21 nuclei; *P* < 0.0001), confirming that CO formation is globally increased (Figure 5A and B).

We next pursued a genome-wide next-generation sequencing (NGS) approach. To this end, we crossed heterozygous *atm-2* plants (in the Columbia-0 background; Col-0) to heterozygous *atm-1* plants (in the Wassilewskija background; Ws; Garcia et al., 2003) to yield F_1_ hybrid plants that either lacked (*atm-2*/*atm-1*) or expressed functional ATM (*ATM*/*ATM*), compatible with a sequencing-based recombination screen (Rowan et al., 2015; Supplemental Figure S5). To assess potential accession-specific differences in response to the loss of functional ATM with respect to class I CO rates, we counted HEI10 foci in Col-0, Ws, and F_1_ plants either mutated or wild type at *ATM*. HEI10 is an E3 ubiquitin ligase known to promote interference-sensitive CO formation and has been widely used as a proxy for class I CO numbers (Chelysheva et al., 2012; Jahns et al., 2014; Lambing et al., 2015; Kurzbauer et al., 2018). Whereas HEI10 foci numbers significantly differed depending on the accession, they were independent of the allelic status of *ATM* (Supplemental Figure S6). Individual F_1_ plants were used as pollen donors for backcrossing to one of the original accessions (Col-0) to account for the low fertility of *atm* mutants. We sequenced (Illumina HiSeq2500; 125 bp paired-end sequencing) three control plants (parental *atm-1/ATM* [Ws background]; parental *atm-2/ATM* [Col-0 background], heterozygous F_1_ [*ATM*^Col-0^/*ATM*^Ws^]) at high overall coverage (31- to 35.5-fold) to extract all single nucleotide polymorphism (SNP) differences between the two accessions. After stringent quality control, we identified 457,591 SNP positions that were included in the analysis. We randomly selected 81 SNP positions for PCR amplification of about 200–400 bp of sequence around each SNP and performed Sanger sequencing to confirm SNP identity and presence in heterozygous Col-0/Ws F_1_ plants. We confirmed correct SNP assignment for 76 out of 81 positions (94%). Subsequently, we sequenced the genomes of the F_2_ plants resulting from the backcross to identify the allelic status at each SNP, thus allowing us to infer the sites of recombination during meiosis in the previous generation. We used the bcftools consensus caller (Li et al., 2009; Li, 2011) to analyze the zygosity of SNPs genome-wide in 36 wild-type and 45 mutant individuals after sequencing with 1.0- to 8.7-fold coverage. We binned 100 adjacent SNPs, determined the zygosity of each bin and used zygosity switches between bins to identify CO events. On average, we counted 5.2 ± 1.6 COs in wild-type and 5.8 ± 1.6 COs in *atm* mutant plants (Figure 5C). Whereas CO numbers did not significantly differ between wild-type and *atm* mutant plants when comparing all or just the shorter chromosomes 2 and 4 (1.6 ± 1 and 1.6 ± 1.1; *P* = 0.9332) that bear the nucleolus organizing regions (NORs) and generally form fewer class II COs (Lam et al., 2005), CO numbers did significantly increase on the longer chromosomes 1, 3 and 5 of *atm* mutants (3.6 ± 1.3 and 4.2 ± 1.3; *P* = 0.0395; Figure 5D). In line with the data obtained in defined intervals, the occurrence of chromosomes with multiple COs increased in *atm* mutant plants (from 27.2% to 32.4%; Supplemental Figure S7A and B). The overall distribution of CO frequencies in wild-type and *atm* mutant plants was very similar (Supplemental Figure S7C). Displaying the CO frequency according to the proportional length of all chromosome arms also revealed a very similar pattern between wild-type and *atm* plants, with an increase in CO frequencies closer to the telomeres in *atm* mutants (Figure 5E).

### Additional COs in *atm-2* mutants depend on class II CO mediators

**Figure 6:**
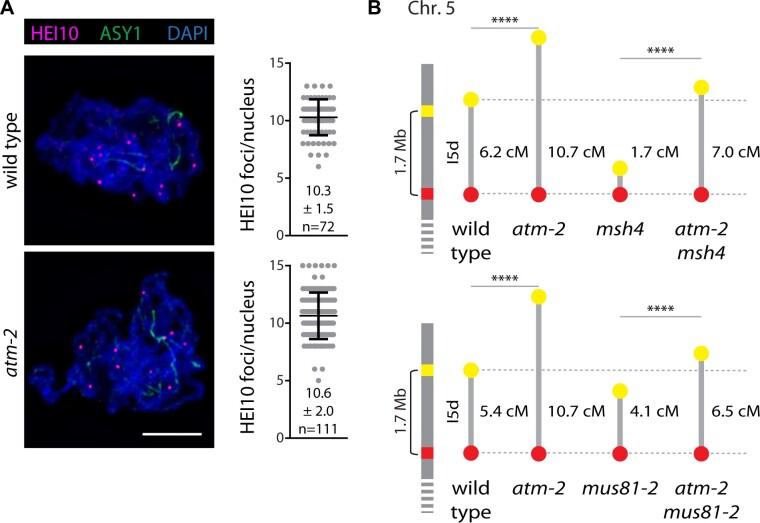
ATM limits non-interfering COs. A, HEI10 foci numbers are not affected by loss of ATM (*P* = 0.2173). Spreads of PMCs were stained for the axial element protein ASY1 (green) and the E3 ubiquitin ligase HEI10 (magenta). Numbers of HEI10 foci were determined in pachytene nuclei. Chromatin was stained with DAPI (blue). Bar: 5 μm. Error bars represent SDs. B, Recombination increase in *atm-2* does not depend on MSH4 but to a certain extent on MUS81. CO frequencies in one interval on chromosome 5 (I5d) were analyzed (****P ≤ 0.0001).

In order to identify the CO pathway responsible for the additional COs observed in *atm-2* mutant plants, we performed immunohistochemical staining of the class I CO marker HEI10. We counted similar numbers of HEI10 foci in wild-type (10.3 ± 1.5; *n* = 72 nuclei) and *atm-2* mutant meiocytes (10.6 ± 2.0; *n* = 111 nuclei; *P* = 0.2173) at the pachytene stage, suggesting that the interference-sensitive class I CO pathway is unaffected in the absence of functional ATM (Figure 6A). These results were corroborated by analyzing the *atm-1* mutant allele (Supplemental Figure S6). Localization of class I COs along chromosomes appeared normal; we observed no clustering of HEI10 foci (which would indicate a genuine loss of interference; Jahns et al., 2014) in wild-type (Col-0 and Ws), *atm-2* or *atm-1* plants.

To further investigate the involvement of different CO pathways, we obtained recombination data in the *msh4* mutant background. MSH4 (MutS HOMOLOG 4) is an essential mediator of class I CO formation and its absence leads to a complete loss of class I COs (Higgins et al., 2004). Only complete tetrads can be evaluated reliably when assessing CO formation in two neighboring intervals, but the *atm-2 msh4* double mutant has severe meiotic defects and produces very little viable pollen, leading to strongly reduced fertility (Supplemental Figure S8). We, therefore, analyzed single pollen grains (instead of tetrads) from four to six individual plants per genotype from a segregating population and focused on only one interval on chromosome 5 (I5d). Recombination rates for wild type (6.2 cM, *n* = 4,382 pollen grains) and *atm-2* (10.7 cM, *n* = 2,723 pollen grains) were collected in parallel and found to be very similar to the ones previously obtained with tetrads. Meiotic recombination was significantly enhanced in the *msh4 atm-2* double mutant (7.0 cM, *n* = 1,685 pollen grains) compared to the *msh4* single mutant (1.7 cM, *n* = 4,038 pollen grains; *P* < 0.0001; Figure 6B). We hypothesize that the proportionally stronger increase of recombination events comparing *msh4* and *msh4 atm-2* (4.1-fold) versus wild type and *atm-2* (1.7-fold) is most likely due to the unchallenged activities of class II CO factors like MUS81 (Berchowitz et al., 2007) or FANCD2 (Kurzbauer et al., 2018) in the *msh4* mutant background. Taken together, these results indicate that the class I CO pathway contributes very little, if at all, to the recombination increase observed in *atm-2* mutants.

We also tested whether MUS81, a mediator of interference-insensitive class II COs, affected recombination rates in *atm-2* plants. As above, we analyzed single pollen grains due to the severely compromised fertility of the double mutants (Supplemental Figure S8; Kurzbauer et al., 2018). We manually screened 11,978 wild-type, 5,405 *atm-2*, 9,245 *mus81-2*, and 4,130 *atm-2 mus81-2* pollen grains from four to six individual plants per genotype of a segregating population. While meiotic recombination was significantly enhanced in *mus81-2 atm-2* double mutants (6.5 cM) compared to *mus81-2* single mutants (4.1 cM, *P* < 0.0001), the proportional increase was small (1.6-fold; Figure 6B). The analysis above already excluded additional class I COs as a source for the elevated recombination rates in *atm-2* mutants. To confirm that class I COs are unaffected in *mus81-2 atm-2* mutants, we also analyzed HEI10 foci numbers and determined them to be at wild-type-like levels (10.96 ± 2.4 foci in *atm-2 mus81-2*; *P* = 0.4859, *n* = 26 nuclei). We, therefore, conclude that not only MUS81, but also further class II CO mediators like FANCD2 (Kurzbauer et al., 2018), are responsible for the elevated recombination rates in *atm-2* mutant plants, while class I CO factors are not involved.

### Elevated CO numbers in *atm* mutants are correlated with increased SC length and width and shorter chromatin loops

**Figure 7:**
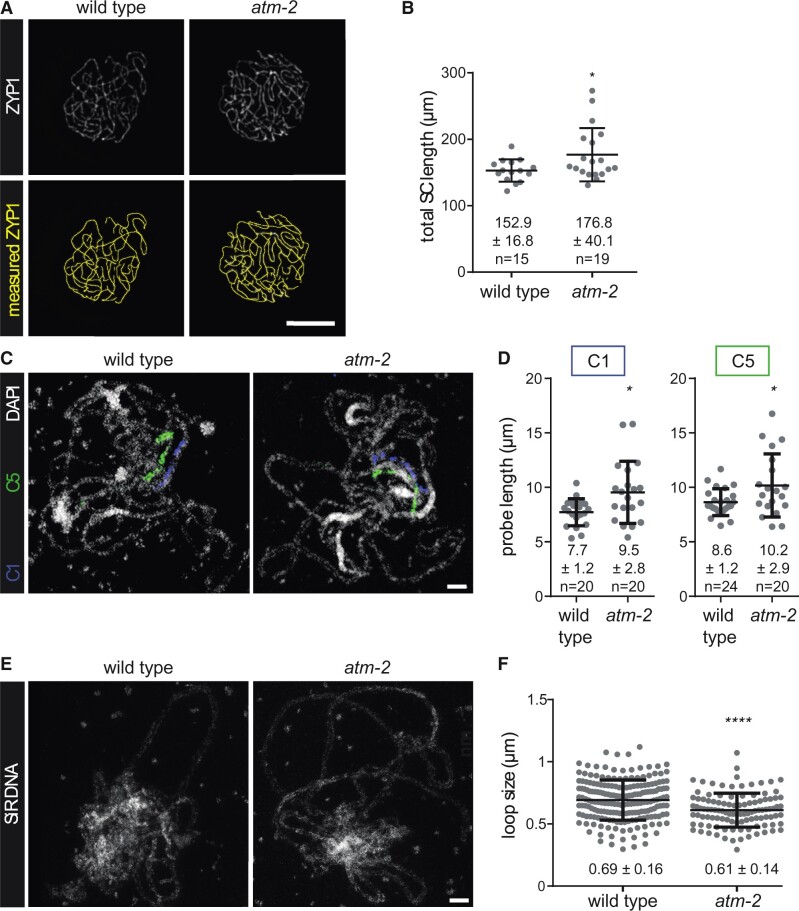
Total and local SC length increase in *atm-2* mutant plants, accompanied by a decrease in chromatin loop size. A, Spreads of PMCs were stained for the SC protein ZYP1 and total SC length measured in pachytene nuclei. Bar: 5 μm. B, SC length increases in *atm-2* mutant plants (*P* = 0.039). C, Two chromosomal regions were painted using continuous FISH probes, and their lengths were determined in projections of 3D-SIM images. Bar: 2 μm. D, The physical length of both probes (C1 spans 2.9 Mb on chromosome 1, C5 spans 3.1 Mb on chromosome 5) increases in the *atm-2* mutant (C1: *P* = 0.0122; C5: *P* = 0.0228). E, Acid spread pachytene nuclei were stained with SiR DNA and imaged on a STED nanoscope. Loop size was measured. Bar: 2 μm. F, Loops are shorter in the *atm-2* mutant than in the wild type (196 measurements in 12 wild-type nuclei; 108 measurements in eight mutant nuclei; *P* < 0.0001). Error bars represent SDs.

Previous studies reported a positive correlation between recombination frequency and SC length (Kleckner et al., 2003; Giraut et al., 2011; Ruiz-Herrera et al., 2017; Wang et al., 2017; Wang et al., 2019a, 2019b). Since our analyses showed increased recombination frequencies in *atm* mutants, we investigated whether a concomitant increase in SC length would be observed. Utilizing an antibody directed against the SC protein ZYP1 (Higgins et al., 2005), we determined overall SC length at the pachytene stage on preparations of spread chromosomes. We measured 152.9 ± 16.8 µm (*n* = 15 nuclei) in wild-type plants and observed a significant increase to 176.8 ± 40.1 µm (*n* = 19; *P* = 0.039) in the *atm-2* mutant (Figure 7A and B). We corroborated these results by measuring the length of two specific chromosomal regions at the pachytene stage, marked by FISH utilizing specific DNA probes. The interrogated regions span 2.9 Mb on chromosome 1 (C1) and 3.1 Mb on chromosome 5 (C5). Region C5 corresponds to the interval I5cd, evaluated for recombination frequency above (Figure 4). For region C1, we measured 7.7 ± 1.1 µm (*n* = 20) in wild-type pachytene nuclei and a significant increase to 9.5 ± 2.8 µm (*n* = 20; *P* = 0.0122) in *atm-2* mutant plants. For region C5, we measured 8.6 ± 1.2 µm (*n* = 24) in wild-type plants and a significant increase to 10.2 ± 2.9 µm (*n* = 20; *P* = 0.0228) in the *atm-2* mutant (Figure 7C and D), thus confirming a general increase in SC length. Using stimulated emission depletion (STED) nanoscopy to observe acid-spread and SiR-DNA-stained pachytene nuclei enabled us to measure chromatin loop size. We determined that the ATM-dependent increase in SC length was accompanied by the formation of shorter loops at the pachytene stage. Loops (for both homologs) spanned 692 ± 161 nm in wild type (196 measurements from 12 wild-type nuclei) and were significantly shorter in the absence of functional ATM (611 ± 136 nm; 108 measurements from eight *atm-2* nuclei; *P* < 0.0001; Figure 7E and F). Using these values, together with total SC length as measured above, we calculated loop density according to (Kleckner, 2006) and estimated 14.3 loops per micron for wild type. Notably, there was no difference when we estimated loop density for the *atm-2* mutant (14.4 loops/micron).

**Figure 8:**
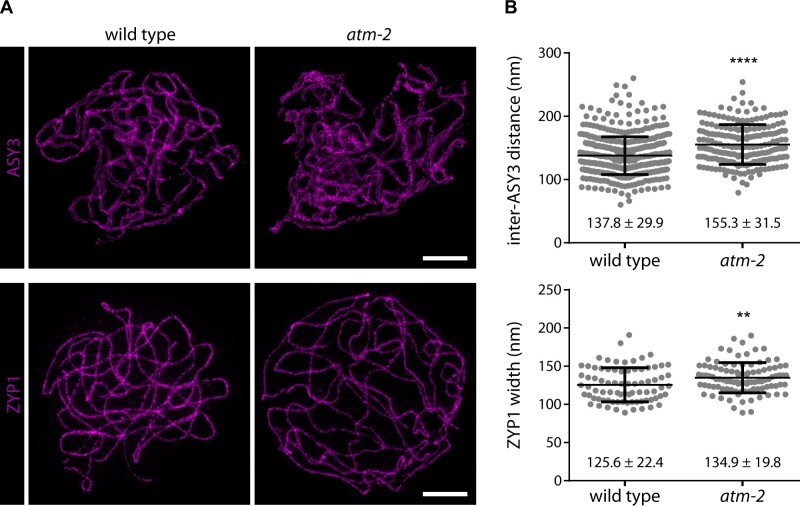
Inter-axis distance and SC width are altered in the *atm-2* mutant. A, Nuclei were stained for the lateral-element-like protein ASY3 and the transverse filament protein ZYP1. The axis and SC appear unperturbed in *atm-2* mutant plants. Bars: 2 μm. B, Inter-lateral-element distances were measured between the centers of ASY3-labeled stretches along pachytene chromosomes and found to be increased in the absence of functional ATM (488 measurements in 12 wild-type nuclei; 204 measurements in nine mutant nuclei; *P* < 0.0001). Similarly, the width of ZYP1 filaments increased in *atm-2* mutant plants (79 measurements in six wild-type nuclei; 99 measurements in five mutant nuclei; *P* = 0.0025). Error bars represent SDs.

We also analyzed the axis components ASY1 and ASY3 (ASYNAPTIC 1/3) at the zygotene and pachytene stages. ASY1 is mostly removed from meiotic chromosomes upon chromosome synapsis and only remains prominently stained at the rDNA regions (Sims et al., 2019). ASY3 is then part of the SC as one of the factors building the lateral elements. STED nanoscopy, with an XY resolution between 40 and 60 nm, enabled us to visualize the aligned axes of synapsed homologs. The distance between opposing ASY3 signals (i.e., homologous chromosomes) along pachytene chromosomes was significantly larger in the *atm-2* mutant (155.3 ± 31.5 nm) compared to wild type (137.8 ± 29.9 nm; 488 measurements in 12 wild-type nuclei; 204 measurements in nine mutant nuclei; *P* < 0.0001; Figure 8). We confirmed these results using a second *atm* mutant allele (128.6 ± 29.8 nm in Ws wild-type; 226 measurements in six nuclei; 145.2 ± 32.0 nm in *atm-1*; 312 measurements in eight nuclei; *P* < 0.0001; Supplemental Figure S9A and B). This phenomenon specifically depended on the lack of functional ATM, since a mere increase of class II CO numbers by mutation of *FANCM* (Crismani et al., 2012) had no effect (138.1 ± 23.9 nm; 180 measurements in nine *fancm-1* mutant nuclei; *P* = 0.5882 compared to wild type; Supplemental Figure S9C and D). The increase of inter-axis distance was corroborated by measuring the width of the signal generated with an antibody directed against ZYP1 (Higgins et al., 2005), which builds the transverse filaments of the SC. We measured a width of 134.9 ± 19.8 nm in the *atm-2* mutant and a significantly smaller value in wild-type plants (125.6 ± 22.4 nm; 79 measurements in six wild-type nuclei; 99 measurements in five mutant nuclei; *P* = 0.0025; Figure 8). While inter-axes distances differed, the general appearance of the meiotic axis and the SC was otherwise normal in *atm-2* plants (Figure 8; Supplemental Figure S10). Deposition of meiotic cohesin, visualized on meiotic chromosomes by staining for the meiosis-specific cohesin subunit REC8 (Chelysheva et al., 2005; Cromer et al., 2013) was also very similar in *atm-2* and wild-type plants (Supplemental Figure S11).

## Discussion

Our study draws a comprehensive map of ATM function during meiosis. We demonstrate that the plant ATM kinase is needed to limit the number of meiotic DSBs and is required for complete DNA repair. ATM also controls the number of interference-insensitive COs and modulates SC length and width as well as chromatin loop size. The presented results will guide future studies to identify the pertinent phosphorylation targets and regulatory mechanisms.

An earlier study investigating ATM in Arabidopsis demonstrated that the kinase is required for full fertility and that defective meiotic DNA repair is the most probable underlying cause (Garcia et al., 2003). We confirmed here that DNA fragmentation in *atm* mutants exclusively depends on the formation of meiotic DSBs and observed some aberrant DNA repair in the *atm-2* mutant (formation of DNA bridges). This result indicated that some DSBs are not processed in a canonical manner when ATM is missing. Most DSBs did however appear to generate recombinogenic ends loaded with RAD51 and DMC1. Indeed, the number of recombinase foci significantly increased, which may be the result of a surplus of meiotic DSBs or a slowed turnover of RAD51/DMC1 nucleoprotein filaments. Two independent experimental approaches determined that meiotic DSB numbers increased in Arabidopsis *atm* mutants. First, blockage of the DNA repair pathway downstream of DSB formation and upstream of recombinase loading resulted in a 2-fold increase in the number of chromosome fragments in *atm* mutants. Second, and corroborating this result, we showed that counting the number of SPO11-1-oligos, which represent the fraction of SPO11-1 proteins that have engaged in a DNA cleavage reaction, also yields a 2-fold increase in *atm* mutants. These findings are in line with the analysis of the meiotic function of ATM in other organisms; an up to 2-fold increase of DSBs was also reported for *atm* (*tel1*) mutants in *S. cerevisiae* (Mohibullah and Keeney, 2017), *Drosophila melanogaster* (Joyce et al., 2011), and *C. elegans* (Li and Yanowitz, 2019). In mouse, an 11-fold increase has been determined (Lange et al., 2011). We, therefore, conclude that ATM is a conserved, negative regulator of meiotic DSB formation.

An increase in DSBs may result in additional CO formation. We analyzed meiotic recombination in four genetic intervals located on two different chromosomes utilizing a pollen-based tester system (Berchowitz and Copenhaver, 2008) and demonstrated that recombination rates are significantly elevated in *atm-2* mutant plants. Interference appeared disturbed, but the number and distribution of HEI10 foci, a proxy for class I COs, was unchanged in *atm-2* mutant plants. This observation indicates that the class I CO pathway is unaffected. Conversely, elevated CO numbers in the *atm-2* mutant depended on class II CO mediators. We demonstrated that a significant number of the additional DSBs were converted into MUS81 (class II CO)-dependent COs. Accordingly, MUS81 became a crucial factor in the *atm-2* mutant background, contributing significantly to its (residual) fertility. Recombination increase in *atm-2 mus81-2* double mutants was not completely abolished, likely due to other class II CO factors, such as FANCD2, still being present and active (Kurzbauer et al., 2018). Mutation of *ATM* furthermore strongly increased recombination rates in an *msh4* mutant background, lending further support to the findings discussed above that class II CO factors, rather than class I, are highly active in the absence of the ATM kinase. We also observed an increase of complex recombination events in *atm-2* mutant plants. While we cannot conclusively show that they originate from locally clustered DSBs, as observed in yeast *tel1* mutants (Garcia et al., 2015), their appearance is consistent with this idea. We, therefore, conclude that, in addition to limiting the number of DSBs, ATM is also involved in restricting non-interfering COs and preventing CO-clustering.

We also discovered an ATM-dependent increase in chiasmata, using a cytological approach and subsequently performed a genome-wide analysis of recombination. We used a back-crossing strategy to account for the fertility defects observed in *atm* mutants. Our survey revealed elevated recombination in *atm* mutants, yet the observed increase was less pronounced than in all other assays. This apparent moderate increase may be due to a general decrease of COs due to heterozygosity at SNP sites (Drouaud et al., 2013) and clearly reflects an underestimation of complex recombination events (i.e., multi-COs) due to the experimental setup; the backcrossing strategy used to obtain individuals for sequencing makes recombination events appear both less complex and to occur less frequently. Notably, interactions involving several strands that appeared specifically increased in the FTL screen will be resolved into simple CO patterns. In addition, a possible selection for undamaged genomes during gametogenesis, fertilization, and growth of the tester plants yielding only survivors among the *atm* mutants may further affect the results. A previous experiment assaying recombination of phenotypic markers in *atm-1* mutant plants showed that there was no apparent change in meiotic recombination frequencies (Garcia et al., 2003). The phenotypic markers used were separated by more than 13 Mb on chromosome 1, impeding the ability to assay subtle differences or double COs within this region (Perkins, 1949).

It is interesting to compare our results to those obtained in other organisms. In the *S. cerevisiae tel1* mutant, class II COs are also elevated, whereas the number of class I COs increased in mouse strains lacking ATM (Barchi et al., 2008; Anderson et al., 2015). Furthermore, interference between MLH1-dependent COs changed in mouse and the formation of the obligate COs on XY chromosomes was severely perturbed (Barchi et al., 2008). In *C. elegans*, the number of COs is generally reduced in *atm-1* strains (Li and Yanowitz, 2019). The DDR signaling cascade consists of different branches and targets in various organisms; therefore, ATM deficiency may lead to distinct outcomes. For instance, plants lack clear homologs for many downstream regulators like the Checkpoint Kinases CHK1 and CHK2, p53, and MDC1, which act in the signaling cascade triggered by mammalian ATM. Similarly, another phosphatidylinositol 3′-kinase-related kinases family member implicated in DNA repair, DNA-PKcs (DNA-dependent protein kinase), does not appear to be present in plant genomes (Templeton and Moorhead, 2005; Cools and De Veylder, 2009; Yoshiyama et al., 2013; Roitinger et al., 2015).

Elevated CO numbers have been correlated with extended SC length (Kleckner et al., 2003; Giraut et al., 2011; Baier et al., 2014; Wang et al., 2017; Wang et al., 2019a, 2019b), which in turn is correlated with shorter chromatin loops (Zickler and Kleckner, 1999). Most of the mentioned studies focused on class I COs, but the same correlation was also observed when measuring total CO numbers (Giraut et al., 2011). We, therefore, measured SC length by tracing the SC transverse filament protein ZYP1 (Higgins et al., 2005; Osman et al., 2006), or by highlighting defined regions on chromosomes 1 and 5 by chromosome painting in pachytene nuclei and detected a significant increase in local and global SC length in *atm-2* mutant plants. Employing STED nanoscopy allowed us to measure chromatin loop size, which was indeed shorter, as suggested by the increase in SC length. Loop density, however, was not affected by mutation of ATM. While density values were similar to those estimated for mouse and yeast (Kleckner, 2006; Patel et al., 2019), they are only an approximation, since loop size and SC length were measured using different spreading techniques and in independent experiments. Absolute values might change when using different means of measuring or calculating. Nanoscopy was also used to determine the width of the SC, either by measuring the distance between the homologous chromosomes stained for the lateral element protein ASY3, or the breadth of the transverse filament ZYP1, and showed that the SC is wider in *atm-2* mutant plants. Since SC width was not affected by a mere increase of class II COs as seen in the *fancm-1* mutant, this effect appears to specifically depend on ATM function. Divergence in generally highly conserved SC width has so far only been described as one of several morphological differences of chromosomes between sexes in mouse and the silk moth *Bombyx mori*, although the underlying mechanism is still unknown (von Wettstein et al., 1984; Agostinho et al., 2018). Deletion of parts of the yeast Zip1 protein coiled-coiled region artificially caused a decrease of SC width (and lower recombination frequencies), whereas insertions led to increased width (Sym and Roeder, 1995; Tung and Roeder, 1998). The sequences of the two Arabidopsis ZIP1 homologs, ZYP1A and B, contain several SQ sites (three in ZYP1A arranged in a putative S/TQ cluster domain; within 37 amino acids) within their coiled-coil domains, making one or even both likely targets for modification and control by the ATM kinase. It has been suggested that modulating SC width will affect the efficiency or stability of homolog invasion events (von Wettstein et al., 1984; Agostinho et al., 2018; Cahoon and Libuda, 2019). In contrast to the disruptions and gaps observed in *atm* mutant mice (Barchi et al., 2008), axis, SC, and cohesion formation (as visualized by ASY1, ASY3, ZYP1, or REC8, respectively), appear unperturbed in mutant plants. The meiotic chromosome axes are likely involved in CO control by providing the connective base for a signal that governs the distribution of COs and possibly also DSBs (reviewed in Zickler and Kleckner, 2015, 2016). Our experimental data are in agreement with this idea and propose that ATM (kinase activity) is involved in altering meiotic chromosome axis and SC characteristics, thereby influencing DSB and CO formation and patterning. Further, our data identify ATM as affecting the organization of the axis/SC without being a structural component thereof.

Having charted the involvement of ATM during meiotic progression, the next step will be to identify the targets that are directly involved in the affected meiotic processes. We previously performed a phosphoproteome-wide screen for ATM and ATR targets following DNA damage in somatic tissues (Roitinger et al., 2015). We identified a large number of ATM/ATR dependent targets, including proteins relevant for meiotic progression and DNA repair, among them cohesin regulators (e.g., PDS5, WAPL), DNA repair factors (e.g., MRE11, LIGASE IV, PCNA), or histones (H2AX, H2AW7; Roitinger et al., 2015; Lorkovic et al., 2017). ATM and ATR kinases phosphorylate proteins at S/TQ consensus motifs (Kim et al., 1999), which has been exploited to describe and characterize meiotic ATM/ATR targets in the mouse (Fukuda et al., 2012). The meiotic axis protein HORMAD1 and the cohesin subunit Structural Maintenance of Chromosomes 3 (SMC3) have been shown to be phosphorylated in a DSB-dependent manner at SQ sites (Fukuda et al., 2012) and the presence of the cohesin cofactor Precocious Dissociation of Sisters 5 (PDS5) is crucial for wild-type SC length in the mouse (Viera et al., 2020). In yeast, the HORMAD1 orthologue, Hop1, and the DSB cofactor Rec114, have been identified as Tel1 (ATM) and Mec1 (ATR) targets that are phosphorylated in a DSB-dependent manner (Carballo et al., 2008; Carballo et al., 2013). However, impeding phosphorylation of S/TQ sites in Rec114 does not fully recapitulate a *tel1* deletion with respect to DSB regulation, indicating that further ATM targets are involved (Mohibullah and Keeney, 2017). A proteomics study found that the *Brassica oleracea* axis proteins ASY1 and ASY3 are extensively phosphorylated at consensus ATM target sites (Osman et al., 2018) and a similar pattern can be expected for the closely related plant Arabidopsis. Further research is needed to fully understand the exact mechanistic effects of ATM on meiosis in different organisms.

Taken together, we demonstrate that the role of ATM during meiosis extends beyond that of being a mere sensor and transducer of DNA damage. ATM is an essential component of a regulatory network that controls meiotic DSB and CO formation and SC organization.

## Materials and methods

### Plant growth conditions

Plants were grown under long-day conditions (16 h light, 8 h dark, 21°C; 60% humidity, 15,550 lux, T5 Tube illumination) at the Plant Sciences Facility at Vienna BioCenter Core Facilities (VBCF), member of the Vienna BioCenter (VBC), Austria.

### Mutant plant lines

The following mutant plant lines were used: *atm-1* (Wassilewskija accession; INRA line; Garcia et al., 2003), *atm-2* (in the Col-0 background; SALK_006953; Garcia et al., 2003), *com1-1* (SALK_061706; Uanschou et al., 2007), *fancm-1* (EMS mutant; Crismani et al., 2012), *lig4-4* (SALK_04427; Heacock et al., 2007), *msh4* (SALK_136296; Higgins et al., 2004) *mus81-2* (SALK_107515; Hartung et al., 2006), *spo11-1-2* (EMS mutant; Grelon et al., 2001), and *spo11-2-3* (GK_749C12; Hartung et al., 2007). Confirmation of genotypes was performed by PCR using the following primers (see Supplemental Table S5): *atm-1*: ATM104, ATM123, and TAG3; *atm-2*: ATM-F1, ATM-R1, and SALK LBc1; *com1-1*: Com1dn, Com1up, and SALK LBc1; *fancm-1*: FANCM_CAPS_F, and FANCM_CAPS_R, *MboI* digestion; *lig4-4*: LIG4-8, LIG4-9, and SALK LBa1; *msh4*: MSH4_F1, MSH4EXP_R1, and SALK LBa1; *mus81-2*: Mus81_LP, Mus81_RP, and SALK LBa1; spo11-1-2: spo11-1_allele_new, spo11-1_pin_up, *Vsp*I digestion; *spo11-2-3*: Spo11-2_down, Spo11-2_up, and GABI-1.

### Seed per silique counts

All analyzed plants were grown side-by-side. Mature but still green siliques originating from the 5^th^ to the 30^th^ flower of main shoots were harvested into 96% EtOH and left at room temperature for 1–3 d for destaining. Ethanol was exchanged several times until the tissue was clear of chlorophyll and seeds inside siliques were counted manually under a dissection microscope.

### Cytology

Spreads of PMCs for DAPI/SiR DNA staining and chromosome painting were prepared as described (Vignard et al., 2007). SiR DNA (Spirochrome; 1:100 in PBS) was applied for 20 min in a humid atmosphere at room temperature before mounting with ProLong™ Glass Antifade Mountant (Invitrogen). Control plants for fragment counts were exposed to 100 Gy ionizing radiation (25 Gy min^−1^) from a Co^60^ source and inflorescences fixed 25 h after treatment.

Prophase spreads of PMCs for cytological detection of proteins were prepared from primary inflorescences of at least five plants per genotype as described (Kurzbauer et al., 2012; Sims et al., 2020). The following primary antibodies were used, as described previously: anti-HEI10 raised in rabbit (1:150; Chelysheva et al., 2012), anti-ASY1 raised in rabbit (1:500; Armstrong et al., 2002), rat (1:500; Higgins et al., 2004), or guinea pig (1:10,000; Sims et al., 2019), anti-ASY3 raised in rabbit (1:300; Ferdous et al., 2012), anti-RAD51 raised in rat (1:500; Kurzbauer et al., 2012), anti-DMC1 raised in rabbit (1:20; Chelysheva et al., 2007), anti-REC8 raised in rabbit (1:250; Cromer et al., 2013), and anti-ZYP1 raised in rat (1:500; Higgins et al., 2005). The secondary antibodies used were as follows: goat-anti-rabbit conjugated to Alexa Fluor 488 (1:400; Invitrogen; 10236882), donkey-anti-rat conjugated to Alexa Fluor 488 (1:400; Invitrogen; 10123952), goat-anti-guinea pig conjugated to Alexa Fluor 488 (1:400; Invitrogen; 10193752), goat-anti-rabbit conjugated to Alexa Fluor 568 (1:400; Invitrogen; 10032302), goat-anti rat conjugated to Alexa Fluor 568 (1:400; Invitrogen; 10748034), goat-anti-rabbit conjugated to STAR RED (1:100; Abberior, STRED-1002), anti-rat conjugated to STAR RED (1:100; Abberior; N.A.), anti-rabbit conjugated to STAR ORANGE (1:100; Abberior, STORANGE-1002), and goat-anti-rat conjugated to STAR 580 (1:100; Abberior, ST580-1007).

FISH probes for chromosome painting were prepared and labeled by incorporation of Cy5-dUTP or Alexa488-dUTP (Fisher Scientific) during nick translation as described previously (Pecinka et al., 2004). The C1 probe pool encompasses all available BACs from F11P17 to F12B7, spanning 2.9 Mb on chromosome 1. The C5 probe pool encompasses all available BAC clones from MOJ9 to F1N13, spanning 3.1 Mb on chromosome 5.

For chromosome painting, slides containing acid-spread PMCs were washed in 2× saline sodium citrate (SSC) buffer (5 min, room temperature; 20× SSC: 3 M NaCl, 0.3 M Na_3_-citrate), and subsequently dehydrated in an ethanol series (70% [v/v], then 90%, then 100% ethanol, 2 min each). The dried probe was dissolved in 12 µL hybridization mix (50% formamide, 2× SSC, 10% dextrane sulfate) per slide and dissolved for 1 h at 37°C under constant shaking. The probe was denatured at 95°C for 5 min, centrifuged quickly and kept on ice for at least 10 min until further use. 12 µL of probe were added to each slide, covered with a cover glass and sealed with rubber cement. Denaturation was performed on a hot plate at 80°C for 2 min and hybridization in a humid atmosphere overnight at 37°C. The next day, coverslips were removed and slides washed at 42°C, once for 3 min in 2× SSC, once for 5 min in 50% formamide in 2× SSC, once for 10 min in 50% formamide in 2× SSC, and twice for 5 min each in 2× SSC. Finally, 15 µL of DAPI (2 µg/mL) in Vectashield was added, coverslips applied, and slides sealed with nail polish.

Imaging of DAPI-stained PMC spreads and immunostained nuclei were performed with a conventional fluorescence microscope (Zeiss Axioplan) and appropriate filters. Z-stacks were acquired in 100 nm intervals, deconvolved using AutoQuantX software, and are presented as projections done with HeliconFocus software. PMCs hybridized with probes against C1 and C5 have imaged on an Elyra PS.1 microscope system with the ZENblack (Carl Zeiss GmbH) software (Weisshart et al., 2016). Maximum intensity projections of nuclei were calculated via the ZEN software. Probe length was measured using the ZEN software. ASY1, ASY3, ZYP1, REC8, and SiR-DNA-stained nuclei were imaged on an Abberior STEDYCON system with a theoretical X-Y resolution limit of 60 nm for orange (STAR 580 or STAR ORANGE) and 40 nm for far-red dyes (STAR RED, SiR-DNA).

### Foci and fragment counting

Recombinase foci, HEI10 foci, and chromosomal fragments were counted manually with the help of the Count tool in Adobe Photoshop (CC 2018). Fragment numbers were determined by counting all DAPI-stained bodies that were significantly larger and brighter than organelles in the organelle band and subsequent subtraction of the number of expected chromosomes (10 in metaphase II and 20 in anaphase II). The described method underestimates the number of total fragments since some may be too small to be detected.

### Measuring SC widths and chromatin loop size

Unprocessed STED images of Lipsol-spread nuclei were used to measure the distance between the centers of ASY3-labeled chromosome axes in pachytene nuclei. Only chromosomal stretches with the SC in assumed frontal view (local maximum distance between ASY3 stretches) were considered. The width of the signal of the transverse filament protein ZYP1 was determined by measuring the distance between the first and last signal-containing pixel along a line perpendicular to the chromosomal thread. ASY3 and ZYP1 were visualized separately with secondary antibodies conjugated to STAR RED dyes to achieve the highest possible resolution. Chromatin loop size was measured in SiR-DNA-stained acid-spread pachytene nuclei analogous to ZYP1-width measurements. All measurements were performed in FIJI (Schindelin et al., 2012).

### Genome-wide chiasma counting

Fixation, spread preparation, FISH, and chiasma counts were performed as described previously (Sanchez Moran et al., 2001; Lopez et al., 2012; Armstrong, 2013; Kurzbauer et al., 2018), except that locked nucleic acid (LNA) probes were used (5S rDNA: 5′-TYE563-CAAGCACGCTTAACTGCGGAGTTCTGAT-3′; 45S rDNA: 5′-TYE655-GGTCCGAGGATTTGTCGACCAG-3′; both produced by Exiqon). Imaging was performed with a conventional fluorescence microscope (Zeiss Axioplan) and appropriate filters. Localization of 45S and 5S rDNA probes, together with chromosome morphology, allowed for the unambiguous identification of chromosomes and chiasma counting.

### Determination of genetic distances and interference ratios

Genetic distances and interference were determined as described before for pollen tetrads (Berchowitz and Copenhaver, 2008) and single pollen grains (Yelina et al., 2013). The following lines were used: FTL1506, FTL1524, and FTL965 for analysis of chromosome 2 and FTL1963, FTL1143, and FTL2450 for analysis of chromosome 5. Pollen was manually evaluated using a widefield microscope with appropriate filter sets (triple band filter: 422/503/572 HC; single filters: CFP [438/24; 465/30], YFP [513/17; 559/34], and Rhodamine–TexasRed–PE [542/27; 585/29]).

### Generation of a transgenic plant line expressing *SPO11-1-Myc18*

A *SPO11-1* expression construct was generated to express an 18× Myc-tagged genomic clone of Arabidopsis *SPO11-1* under control of its native promoter. The putative 5′ untranslated region (5′ UTR), entire genomic coding region, and putative 3′ UTR were amplified as two fragments from Col-0 genomic DNA using high fidelity Novagen KOD DNA polymerase (Merck Millipore). 5′ UTR and coding region were amplified as a 3,765 bp *Pst*I/*Bam*HI fragment using primers gAtSPOdn and gAtSPO_STOP up. The reverse primer introduces a *Sma*I blunt end restriction site in frame (encoding Pro + Gly) upstream of the translation stop codon used for C-terminal tagging. The 3′ UTR was amplified as a 447 bp *Sma*I/*Bam*HI fragment using primers gAtSPO_STOP_dn and gAtSPOup. Following *Taq* DNA polymerase-mediated A-tailing, both fragments were cloned separately into pCR2.1-TOPO (Thermo Fisher Scientific), sequenced, and subcloned into the binary vector pCB302 (Xiang et al., 1999), resulting in the construct pCB302-AtSPO11-1-WT. A 735 bp fragment encoding an 18× Myc epitope tag was inserted as a *Sma*I/*Eco*RV fragment into *Sma*I-linearized pCB302-AtSPO11-1-WT. Orientation of the 18× Myc tag was determined by Sanger sequencing. A clone with the correct orientation was selected, designated pCB302-AtSPO11-1-Myc18, and delivered into Arabidopsis heterozygous *spo11-1-2* mutant plants by Agrobacterium (*Agrobacterium tumefaciens*)-mediated transformation by the floral dip method (Clough and Bent, 1998).

Plants homozygous for *spo11-1-2* and carrying a single-locus insertion of the *SPO11-1-Myc18* transgene were identified during propagation via segregation pattern analysis following selection with glyphosate (three to five spray treatments of 8- to 10-d-old seedlings with first true leaves visible with 150 mg/L BASTA, glufosinate–ammonium 200 g/L, Bayer Crop Science) and additional PCR analysis. *SPO11-1-Myc18* transgenes were detected as 1.2 kb amplicons using primers c-myc_new_dn and gAtSPOup. Homozygous s*po11-1-2* mutant plants were identified via Cleaved Amplified Polymorphic Sequence (CAPS) marker analysis using amplification primers spo11-1_allele_new and spo11-1_pin_up, followed by *Vsp*I digestion. The wild type yields fragments of 1,274 and 820 bp in size. In the presence of the *spo11-1-2* mutation, the digested PCR products yield fragments of 820, 654, and 620 bp. Plant lines homozygous for both the transgene *SPO11-1-Myc18* and the *spo11-1-2* mutation showed full complementation of sterility, thereby demonstrating the functionality of the transgene. The *SPO11-1-Myc18* line was crossed to heterozygous *atm-2* mutant plants and individual plants homozygous for the *SPO11-1-Myc18* transgene and the *atm-2* mutation from the F_2_ population were used for further analysis. Plants homozygous for the *SPO11-1-Myc18* transgene were used as control.

### Arabidopsis protein lysates

Plants of all genotypes were grown in parallel until flowering under long-day conditions and meiotic tissue was harvested in the form of primary and secondary juvenile flower buds. Inflorescences were trimmed of post-meiotic and already open flowers, snap-frozen in liquid nitrogen, and stored at –80°C until further use. For each independent preparation of SIM-TIRF protein extracts (four in total), about 60 mL of inflorescences were used, corresponding to approximately 12 g of wet weight tissue. Inflorescences were disrupted in liquid nitrogen in a Freezer Mill 6870 (SPEX SamplePrep) at 15 cycles/s. In total, seven disruption cycles comprising of 2 min disruption and 2 min cooling each were performed. Plant powder was stored at –80°C for a maximum duration of 24 h until further use. Frozen plant powder was resuspended in 5 mL of 2× Ferrando buffer (80 mM Tris-HCl; 16% glycerol; 1.6 mM EDTA; 0.8% NP-40; 1.6 mM DTT; pH 7.5) supplemented with 2× Protease Inhibitor Cocktail (Roche Complete Mini EDTA free), pepstatin A (20 µg/mL), and phenylmethanesulfonyl fluoride (PMSF; 2.5 mM). After 30 min incubation on ice, lysates were cleared by centrifugation at 14,000*g*, 4°C for 10 min.

### Yeast meiotic time courses

For the generation of highly synchronized yeast cultures (*S. cerevisiae*; SK1) a yeast meiotic time course was performed, as described previously (Wu and Burgess, 2006) with the following modifications: 5 mL of YPD+AUT (1% yeast extract, 2% bacto peptone, 2% glucose, 0.01% adenine sulfate, 0.01% uracil, 0.01% L-tryptophan) was inoculated with a single yeast colony and incubated at 30°C for 24 h. Cells were harvested by centrifugation at 3,250*g* for 3 min and washed twice in freshly prepared yeast presporulation medium (SPS; 0.5% yeast extract, 1% yeast peptone, 0.17% yeast nitrogen base, 1% potassium acetate, 0.5% ammonium sulfate, 1.02% potassium biphthalate; pH 5.5). 50 mL of SPS was inoculated at an initial density of OD_660_ = 0.1, supplemented with 1:1,000 volume of 1% PGG antifoam, and incubated at 30°C and 200 rpm for 13–18 h. Cells were harvested at OD_660_ = 1.2 and washed twice in prewarmed yeast sporulation medium (SPM; 1% potassium acetate; 0.002% uracil, 0.002% L-tryptophan, 0.002% L-histidine, 0.002% L-arginine, 0.003% L-leucine). The pellet was resuspended in 50 mL SPM, supplemented with 1:1,000 volume of 1% PGG antifoam, and incubated at 30°C. Meiotic progression was monitored by harvesting cell aliquots at time points 0, 3, 4, 5, 6, 8, and 9 h post-induction, followed by fixation in 96% ethanol for 1 h, staining with DAPI (0.2 µg/mL) and analysis by fluorescence microscopy. For protein extraction for SIM-TIRF experiments, cells were harvested at the peak of Spo11 expression 3.5 h post-induction (Prieler et al., 2005), washed in spheroblasting buffer (1 M sorbitol, 50 mM potassium phosphate buffer, 20% glycerol, 20 mM EDTA; pH 7.5), snap-frozen in liquid nitrogen and stored at –80°C until further use. Used strains are defined in Supplemental Table S4. Material for five experiments was collected from two independent meiotic time courses.

*Schizosaccharomyces pombe* strains were grown overnight at 25°C in standard Yeast Extract Supplemented (YES) medium (5 g/L yeast extract, 30 g/L glucose, supplemented with 0.15 g/L adenine and 0.1 g/L of uracil, L-histidine, L-lysine, and L-leucine), diluted to an OD_660_ of 0.2, further grown in 200 mL to an OD_660_ of 0.5. Cells were washed 3 times with water, resuspended in Pombe Minimal Glutamate (PMG) medium lacking nitrogen and grown under nitrogen starvation for 16 h at 25°C. After washing, cells were resuspended in 200 mL of PMG medium, and meiosis was synchronously induced by inactivation of a temperature-sensitive allele of the Pat1 kinase (*pat1-114*) by shifting the temperature to 34°C. Cells were harvested 4.5 h after induction of meiosis by centrifugation, washed in water, snap-frozen in liquid nitrogen, and stored at –80°C until further use. Used strains are defined in Supplemental Table S4. Material for five experiments was collected from four independent meiotic time courses. Each cell aliquot was obtained from an independent meiotic culture.

### Yeast protein lysates

Cell pellets harvested as described above were resuspended in 600 µL yeast lysis buffer (50 mM HEPES-KOH; 140 mM NaCl; 1% Triton X-100; 0.1% sodium deoxycholate; 1 mM EDTA; pH 7.5), supplemented with protease inhibitor cocktail (Roche Complete Mini EDTA free) and PMSF at 1× and 2.5 mM final concentrations, respectively. Cell numbers were determined by immediately counting 1:1,000 dilutions on a CASY Cell Counter System (CASY Model TT, Roche Diagnostics International). Cell suspensions were incubated on ice for 30 min, supplemented with 0.9 volumes of acid-washed glass beads, transferred to 2 mL screw-cap tubes, and disrupted at 2,500 rpm, in a cooled (0°C) bead homogenizer (Multi-beads Shocker, Yasui Kikai). In total, 10 to 20 disruption cycles alternating between 30 s disruption and 30 s cooling were performed. Alternatively, cells were disrupted at 6.5 m/s in a FastPrep Homogenizer (MP Biomedicals). In total 8 disruption cycles alternating between 45 s disruption and 2 min cooling were performed. Disruption efficiency of greater than 90% was confirmed by brightfield microscopy. Finally, lysates were cleared twice by centrifugation at 20.000 *g*, at 0°C for 20 min.

### Immunoprecipitation, nuclease treatment, and TUNEL of Spo11-oligo-complexes

To detect Spo11/Rec12-oligo complexes, Spo11/Rec12-Myc18 fusion proteins were first enriched by anti-Myc immunoprecipitation (IP). ChIP grade mouse anti-c-Myc 9E11 antibody (Abcam; ab56) was conjugated to magnetic beads in NET2 buffer (50 mM Tris-HCl, 150 mM NaCl, 0.05% Nonidet P-40, pH 7.5). For *S. cerevisiae* experiments, 12 µg antibody was conjugated to 25 µL Dynabeads Protein G (Thermo Scientific, 10004D) and for *S. pombe*, 3.6 µg antibody was conjugated to 25 µL Ademtech Protein G beads. For plant experiments, 24 µg antibody was conjugated to 50 µL Dynabeads Protein G. All beads were conjugated overnight at 4°C and washed 3 times with NET2 buffer. Prior to IP, aliquots of protein lysates of 10 µL (yeast) and 100 µL (Arabidopsis) volume were set aside as input and stored at –80°C. Yeast protein lysates were diluted with nine volumes of 2× IP buffer (30 mM Tris-HCl; 300 mM NaCl; 2% Triton X-100; 0.02% SDS; 2 mM EDTA; pH 8.1; Pan and Keeney, 2009). Arabidopsis protein lysates were supplemented with NaCl to 150 mM final concentration. Tagged Spo11 protein was immunoprecipitated for 3–4 h on a rotating wheel at 4°C. Beads were washed 3 times with NET2 buffer and once with 1× NEB4 buffer (50 mM potassium acetate; 20 mM Tris-acetate; 10 mM magnesium acetate; 1 mM DTT; pH 7.9, New England Biolabs). To remove nonspecific copurified RNA and DNA molecules, samples were treated with 500 ng RNaseA (New England Biolabs), 30 U Nuclease RecJf (New England Biolabs), and 5 U Lambda Exonuclease (New England Biolabs) under vigorous shaking for 45 min at 37°C. After two washes with T50+BSA buffer (10 mM Tris-HCl; 50 mM NaCl; 0.1 mg/mL BSA; pH 8.0) and one wash with 1× NEB4 buffer with 0.25 mM CoCl_2_, 3′- hydroxyl termini of Spo11-attached oligonucleotides were labeled with 500 nM Biotin-14-dATP (Thermo) and 20 U TdT ((New England Biolabs) in 1× NEB4 with 0.25 mM CoCl_2_ under vigorous shaking for 60 min at 37°C. TUNEL labeling was terminated by washing 3 times with T50+BSA supplemented with 40 mM EDTA and once with T50+BSA. Samples were resuspended in 5 µL SDS buffer (500 mM Tris-HCl; 2% SDS; 10 mM EDTA; pH 8.1; Pan and Keeney, 2009), transferred to 0.2 mL PCR tubes, and boiled for 5 min at 99°C in a lid-heated PCR cycler. The solution was transferred to a new tube, snap-frozen in liquid nitrogen, and stored at –80°C for a maximum duration of 24 h until further use.

### Biotin-PEG-5K-SVA coated coverslips

The preparation of biotinylated coverslips for SIM-TIRF microscopy was performed as described (Jain et al., 2012) with the following modifications: standard microscope 24 mm × 55 mm coverslips (#1.5 thickness) were cleaned by sonication for 10 min each in ultrapure water, anhydrous methanol (Sigma Aldrich) and anhydrous acetone (AcroSeal, ACROS Organics). Coverslip surfaces were activated by sonication for 20 min in 1 M potassium hydroxide, rinsed with ultrapure water, and dried by baking for 30 min at 110°C. Coverslips were silanized in an anhydrous reaction mix of 95 mL methanol (Sigma Aldrich), 5 mL glacial acetic acid, and 1 mL [3-(2-Aminoethylamino)propyl]trimethoxysilane 97% (APTMS, Sigma Aldrich). Coverslips were incubated under light protection for 20 min and sonicated for 1 min halfway through the silanization process. Silanization was terminated by washing twice with 500 mL ultrapure water. Silanized coverslips were individually rinsed with ultrapure water and dried by baking for 30 min at 110°C or by blowing dry with N_2_. Dried coverslips were stored for up to 2 months at room temperature (under light protection). Biotinylated coverslips were prepared in small batches due to short shelf life and in pairs to save resources. Per coverslip pair, 16 mg mPEG-5K-SVA (45.7 mM, Laysan Bio, MPEG-SVA-5000), and 0.3 mg Biotin-PEG-5K-SVA (0.86 mM, 0.43% (w/v), Laysan Bio, Biotin-PEG-SVA-5000) were used. For 10 pairs of coverslips, 160 mg mPEG-SVA and 3 mg Biotin-PEG-SVA was weighed under argon inert gas atmosphere and dissolved in 500 µL anhydrous N,N-dimethylformamide (DMF; Sigma Aldrich). Coverslips were lined up in a humid incubation chamber and fitted with 18 mm × 18 mm coverslips on their short edge to act as spacers. The Biotin-PEG-SVA/DMF mix was diluted with an equal volume of 100 mM sodium bicarbonate, pH 8.3, and 100 µL of this reaction mix was immediately applied to silanized coverslips and covered with a second silanized coverslip. Coverslip sandwiches were incubated for 3–4 h under light protection. Biotinylated coverslips were individually rinsed with ultrapure water and their biotinylated side marked with an alcohol-resistant lab marker. After drying with nitrogen, coverslips were stored in an evacuated hybridization bag for up to 4 weeks at –20°C under light protection.

### SIM-TIRF flow chamber pulldown of Spo11-oligo complexes

Parallel lines of two-component twinsil 22 dental cement (picodent) were drawn on a standard microscopy slide using a custom-made stainless-steel grille as a template. A series of 20–30 µL flow chambers were created by placing a functionalized coverslip on the rubber cement lines, with the biotinylated side facing inwards. Solutions were applied at one end of the chamber and withdrawn from the other end using pieces of filter paper. All applied volumes were 100 µL and all incubation steps were performed in a humid incubation chamber at room temperature unless otherwise stated. Protein immobilization and detection were performed as described (Jain et al., 2012) with the following modifications: two separate flow chambers were prepared for parallel detection of total Spo11/Rec12 proteins (protein chamber) and Spo11/Rec12-oligo complexes (oligo chamber). Spo11/Rec12 protein was immobilized via anti-Myc pull-down, biotinylated Spo11/Rec12-oligo complexes were immobilized via NeutrAvidin pull-down. Immobilization of samples from different genetic backgrounds was always performed in parallel and on the same coverslip to account for fluctuation of biotin-labeling efficiencies.

Biotinylated surfaces were coated for 15 min with 200 µg/mL NeutrAvidin (Thermo Fisher Scientific, 31000) in T50 buffer (10 mM Tris-HCl; 50 mM NaCl; pH 8.0) and washed twice with T50. The protein chamber was subsequently coated for 30 min with 10 nM of biotinylated anti-mouse antibody (Biotin Goat anti-Mouse, Thermo Fisher Scientific, 31805) and for 20 min with 10 nM of mouse anti-Myc antibody (Abcam, ab56). All antibodies were diluted in T50+BSA buffer (10 mM Tris-HCl; 50 mM NaCl; 0.1 mg/ml BSA; pH 8.0). Protein chambers were washed twice with T50+BSA after each antibody capture and once with 2× IP buffer (yeast experiments) or 2× Ferrando buffer (Arabidopsis experiments). Input protein aliquots and TUNEL labeled Spo11/Rec12-Oligo IP aliquots were thawed on ice. Yeast protein inputs were diluted 20-fold with 2× IP buffer. Arabidopsis protein inputs were used without further dilution. In both experiments, 50 µL of the solution was added to the protein chamber. In parallel, biotinylated SPO11-oligo IP aliquots were diluted 10-fold in 2× IP buffer and applied to the oligo chamber. All chambers were incubated for 20 min at room temperature and subsequently washed twice with detergent wash buffer (20 mM Tris-HCl, 500 mM NaCl, 2 mM EDTA, 0.1% Triton X-100) and once with T50+BSA. To detect immobilized Spo11 moieties, chambers were incubated for 10 min each with 10 nM of anti-Myc antibody (Rabbit anti-Myc, Gramsch, CM-100) and then with 1 nM of goat anti-rabbit Alexa568-conjugated antibody (Invitrogen; 10032302). All chambers were washed twice with T50+BSA after each antibody application. Finally, the positions of the cement strips were marked on the coverslip, which was removed and placed on a fresh microscopy slide prewashed in T50+BSA. Excess buffer was removed in a drying block, slides sealed with transparent nail polish, and stored at 4°C until further use.

### SIM-TIRF microscopy

SIM-TIRF microscopy was performed on an Olympus inverted microscope with a 100× oil immersion TIRF objective (UApo N 100×/1.49 Oil TIRF Corr) using a 561 nm solid-state laser. Penetration depth was set to 150 nm with laser power at 20%–30%. Images were recorded on a Hamamatsu ImagEM X2 EM-CCD camera (512 × 512 pixels; 160 nm/pixel) with typical exposure of 300 ms and EM gain of 200. Several images were recorded for each genotype and exported as 16-bit TIFF files.

### Signal quantification

Signal maxima (corresponding to single SPO11 or REC12 proteins) were quantified using the ImageJ “Find Maxima” function at a threshold of 6,000 and without counting border maxima. Proportions of meiotically active Spo11-oligo molecules were calculated per genotype as the summed total number of SPO11/REC12-oligo maxima divided by the summed total number of total SPO11/REC12 protein maxima, adjusted for total sample volume.

### Genome-wide recombination analysis by deep sequencing

*atm-1/ATM* (Ws accession) and *atm-2/ATM* (Col-0 accession) mutant plants were crossed, giving rise to fully heterozygous F_1_ progeny. Plants carrying no (*ATM*^Col-0^/*ATM*^Ws^) or both (*atm-1/atm-2*) T-DNA insertions in the *ATM* gene were selected by PCR and their pollen used for backcrosses to Col-0 parents to yield the generation to be sequenced. Genomic DNA was prepared from 1 to 2 young leaves per plant (all grown in parallel) as described in (Kurzbauer et al., 2012). DNA concentration was determined using the Qubit 2 fluorometer and the Quant-iT dsDNA Assay Kit (both Thermo Fisher Scientific) according to the manufacturer’s protocol. 20 ng of DNA was used for library preparation by tagmentation according to (Picelli et al., 2014). Tn5 transposase was provided by the VBCF NGS Unit (www.viennabiocenter.org/facilities). Tagmentation was performed for 7 min at 55°C and purification using AMPure XP beads (Agencourt). Barcoding was performed using the Nextera Index Kit (Illumina) and subsequent purification with AMPure XP beads. Quality control and sequencing (Illumina HiSeq2500; 125 bp paired-end sequencing) were performed by the Next Generation Sequencing Facility at Vienna BioCenter Core Facilities (VBCF), member of the Vienna BioCenter (VBC), Austria. In total, three control plants (*atm-2* wild-type parent, Col-0 accession; *atm-1* wild-type parent, Ws accession, and F_1_ hybrid, Col-0/Ws) were sequenced with 31- to 35.5-fold coverage to define SNPs between accessions and 80 samples with 1.0- to 8.7-fold coverage to define CO events. The bioinformatics analysis was performed as follows.

### Mapping

Paired-end reads were trimmed with trimmomatic (Bolger et al., 2014) to eliminate low-quality read ends and remove adapters (default parameters). The remaining reads were mapped to the Arabidopsis TAIR-10 reference genome (https://www.arabidopsis.org) using NextGenMap version 0.5.2 (Sedlazeck et al., 2013) with default parameters. Subsequently, samtools (Li et al., 2009) were used to select the mapped reads that were in proper pairs (SAM flag 3, -f 3), with a mapping quality ≥ 20 (-q 20).

### SNP calling

SNP calling was performed on data retrieved from the three control plants using the samtools/bcftools pipeline (Li et al., 2009; Li, 2011) to extract all SNP differences between the two accessions. Only “homozygous unique” SNP positions (homozygous in each accession but heterozygous in the F_1_ hybrid) were used for the analysis. A total of 457,591 SNPs (Supplemental Table S6) met the above criteria and were included in the analysis (“informative sites”). The samtools/bcftools pipeline was subsequently used on 80 samples to call SNPs at “informative sites” only by using the parameter-positions to enable application of the bcftools consensus caller.

### Detection of CO breakpoints in recombined individuals

Recombination breakpoints (CO positions) were detected using a modified version of the analysis described in Jagut et al. (2016).

Given the crossing scheme delineated above, distinct outcomes for the presence or absence of recombination events were expected. In the absence of a recombination event, the observation of one out of two possible genotypes in the offspring was expected: fully homozygous Col-0 or fully heterozygous Col-0/Ws chromosomes. However, if recombination occurred during the meiotic division in the F_1_, the CO event should create blocks of homozygosity and heterozygosity along the chromosomes. Identifying the changes in zygosity allows detection of the recombination breakpoints.

Since sequencing coverage varied between samples and SNPs were non-homogeneously distributed along the chromosomes, the use of a binning method increased the power of the test at the sites of interest. Hundred adjacent SNPs were combined into one bin and, traversing along each chromosome, neighboring bins were compared. The zygosity of each bin was determined based on the pooled heterozygosity score (Rubin et al., 2010), calculated by

$$h=\frac{2*R*A}{{\left(R+A\right)}^{2}}$$

where *R* is the number of reads that call for the Col-0 allele (reference) and *A* is the number of reads that call for the Ws allele (alternative). Within a heterozygous chromosomal region, an equal number of reads for each allele (*R* and *A*) is expected and thus the pooled heterozygosity score is expected to be close to 0.5. A homozygous region yields a pooled heterozygosity score close to zero. Bins were classified as homozygous when *h* < 0.05, heterozygous if *h* > 0.45, and undetermined otherwise. Finally, adjacent SNPs for each of the two different bins were defined as the site of the recombination event. The window size between two SNPs depends on how they are spread along the chromosome, with an average of 1,534 bp between two adjacent SNPs that were analyzed.

### SNP confirmation

We randomly selected 81 SNP positions, amplified sequences of about 200–400 bp surrounding them by PCR, and performed Sanger sequencing (Microsynth AG) to confirm SNP identity and presence in heterozygous Col-0/Ws F_1_ plants. Oligonucleotide sequences are given in Supplemental Data Set 1.

### Statistical analyses

All statistical analyses were performed using the GraphPad Prism software version 8.4.3. D’Agostino–Pearson omnibus K2 normality testing was applied to determine if data were sampled from a Gaussian population and could be tested by a parametric T test or required testing by a nonparametric Mann–Whitney U test. Accordingly, unpaired, two-tailed Mann–Whitney U tests were performed on data collected by SIM-TIRF microscopy, fragment counts, SC width measurements, and seed counts. Unpaired, two-tailed *t* tests were applied on all foci counts, chiasma counts, NGS-based CO counts and loop size, and total SC length and probe length measurements. Error bars indicate SDs. Due to the binary nature of the results (recombination or no recombination), differences in genetic distances were analyzed by Fisher’s exact test. Test parameters and results can be found in Supplemental Data Set 2.

### Accession numbers

Sequence data from this article can be found at The Arabidopsis Information Resource (TAIR) under the following accession numbers: *ATM* (At3g48190), *RAD51* (At5g20850), *DMC1* (At3g22880), *COM1* (At3g52115), *SPO11*-*1* (At3g13170), *SPO11-2* (At1g63990), *MUS81* (At4g30870), *MSH4* (At4g17380), *HEI10* (At1g53490), *ZYP1A* (At1g22275), *ZYP1B* (At1g22260), *ASY1* (At1g67370), *ASY3* (At2g46980), *FANCM* (At1g35530), *REC8/SYN1* (At5g05490).

All sequences obtained by deep sequencing datasets were deposited at the Gene Expression Omnibus at NCBI under project PRJNA555773.

## Supplemental data

**Supplemental Figure S1.** ATM is required for full fertility.

**Supplemental Figure S2.** Meiotic progression in *atm-2* mutant plants.

**Supplemental Figure S3.** RAD51 foci numbers are elevated in *atm-1*.

**Supplemental Figure S4.** Quantification of DSB levels.

**Supplemental Figure S5.** Crossing scheme for NGS-based recombination screen.

**Supplemental Figure S6.** The number of class I COs depends on the accession but not on ATM.

**Supplemental Figure S7.** CO frequency and distribution in wild type and *atm* mutant plants.

**Supplemental Figure S8.** Meiotic progression and fertility in *mus81-2*, *atm-2 mus81-2*, *msh4*, and *atm-2 msh4* mutant plants.

**Supplemental Figure S9.** The distance between the lateral elements along synapsed chromosomes increases in *atm-1* mutant plants but is not affected by the *fancm-1* mutation.

**Supplemental Figure S10.** Axis formation is normal in *atm-2* mutant plants.

**Supplemental Figure S11.** Cohesin deposition is normal in *atm-2* mutant plants.

**Supplementary Table S1.** SIM-TIRF data overview.

**Supplementary Table S2.** Raw data of tetrad analysis.

**Supplementary Table S3.** Raw data of I5d single pollen grain analysis.

**Supplementary Table S4.** Yeast strains.

**Supplemental Table S5.** Oligonucleotides used in this study.

**Supplemental Table S6.** SNP distribution along the five Arabidopsis chromosomes.

**Supplemental Data Set 1.** Oligonucleotides used for SNP confirmation.

**Supplemental Data Set 2.** Summary of statistical analyses.

## Supplementary Material

koab045_Supplementary_Data

## Notes

P.S. and M.-T.K. are responsible for conceptualization; M.-T.K., M.P.J., K.T., L.F.P., and P.S. contributed to methodology; L.F.P. and A.v.H. are responsible for software; M.-T.K., M.P.J., L.F.P., I.P.M., K.T., O.K., and V.S. contributed to investigation; V.S. and A.v.H. contributed to resources; M.-T.K., M.P.J., and P.S. contributed to writing; P.S. and A.v.H. contributed to funding acquisition.

The author responsible for distribution of materials integral to the findings presented in this article in accordance with the policy described in the Instructions for Authors (https://academic.oup.com/plcell) is: Peter Schlögelhofer (peter.schloegelhofer@univie.ac.at)
