# Supplementary material for: ATM controls meiotic DNA double-strand break formation and recombination and affects synaptonemal complex organization in plants
Source: Plant Cell. 2021 Feb 5;33(5):1633–56. doi: 10.1093/plcell/koab045 (PMC8254504; doi:10.1093/plcell/koab045)
Supplement: koab045_Supplementary_Data [file koab045_supplementary_data.zip › tpc.00768.2020-s03.pdf]

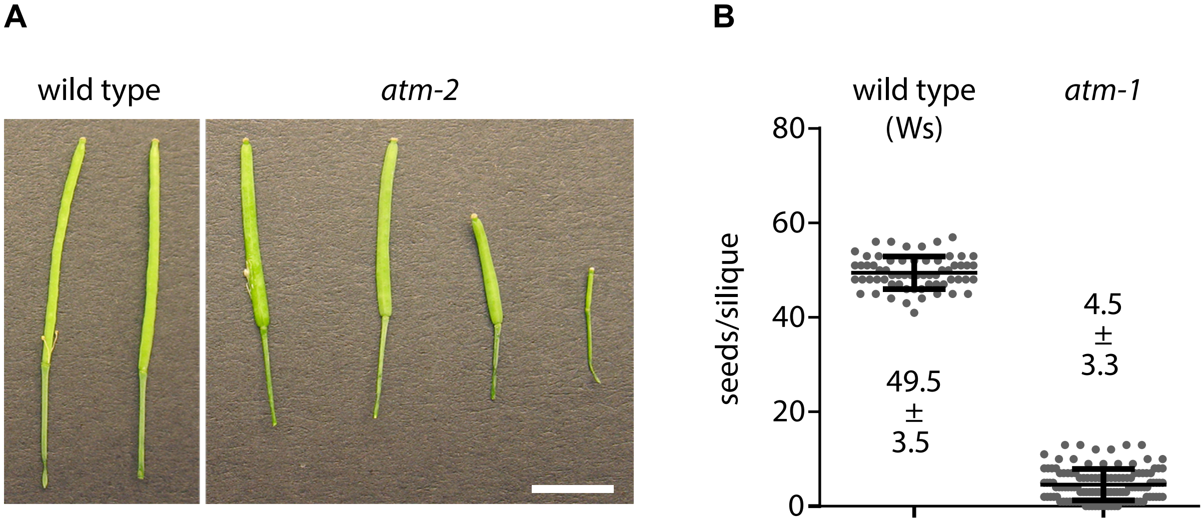

Supplemental Figure 1. ATM is required for full fertility.
(A) Siliques are shorter in *atm-2* mutant plants. Bar: 1 cm.
(B) Similar to *atm-2*, a reduction of fertility is observed in another mutant allele, *atm-1* (4.5 ± 3.3), when compared to its respective Wassilewskija wild type (Ws; 49.5 ± 3.5; n = 124 siliques). Error bars represent standard deviations.
Supports Figure 1.

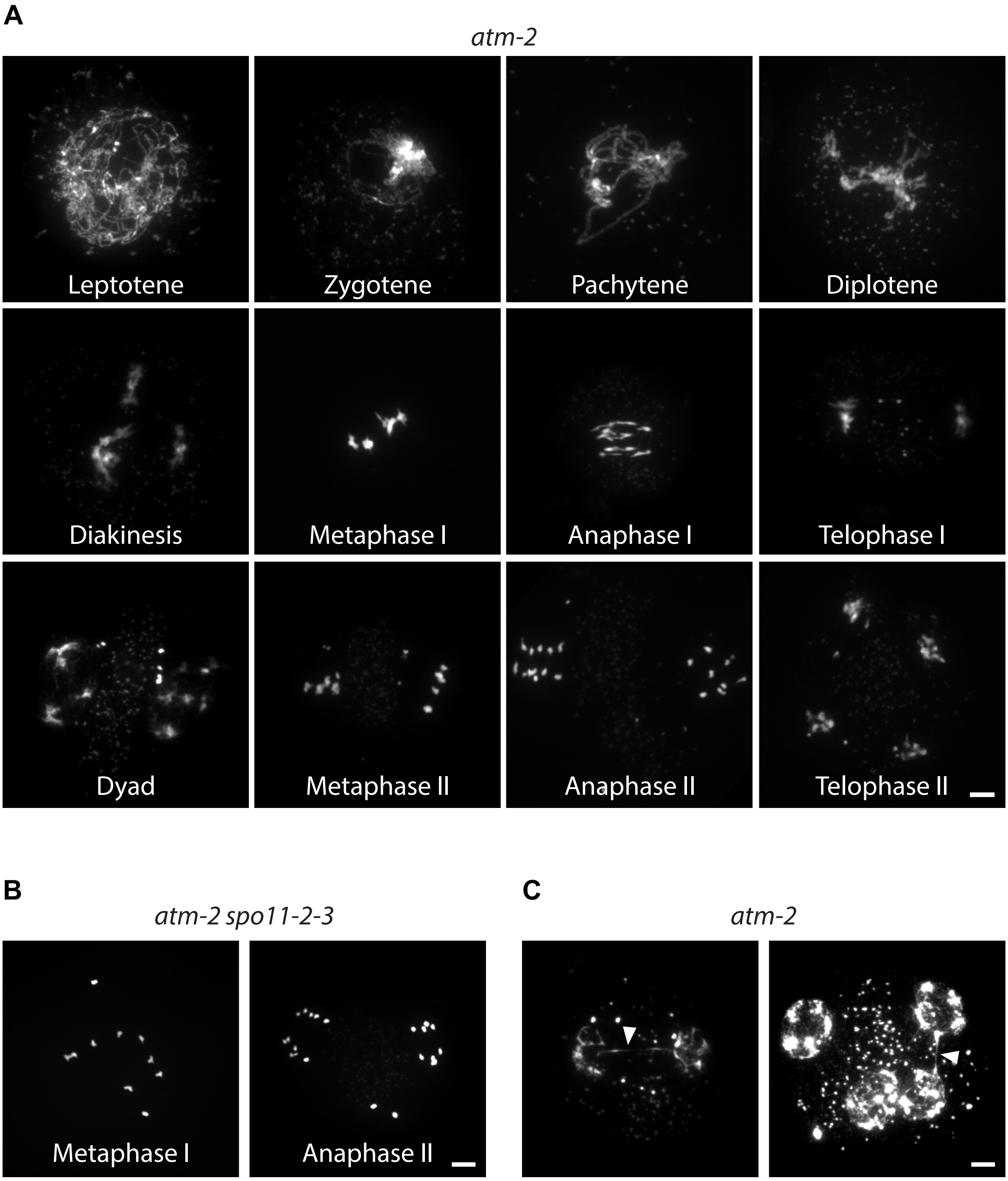

Supplemental Figure 2. Meiotic progression in *atm-2* mutant plants.
(A) Meiosis appears unperturbed up to the pachytene/diplotene stage in the *atm-2* mutant. Diakinesis and metaphase I cells sometimes display entanglements between chromosomes and chromosome fragments become visible at later stages, indicative of DNA repair defects.
(B) Chromosomal fragmentation in *atm-2* depends on SPO11. Chromosomes of pollen mother cells of *atm-2 spo11-2-3* double mutants form univalents in metaphase I and do not display fragmentation at later stages.
(C) Chromosome bridges (arrowheads) were observed in 4 out of 53 *atm-2* nuclei between anaphase I and tetrad stages. Bars: 5 µm.
Supports Figure 1.

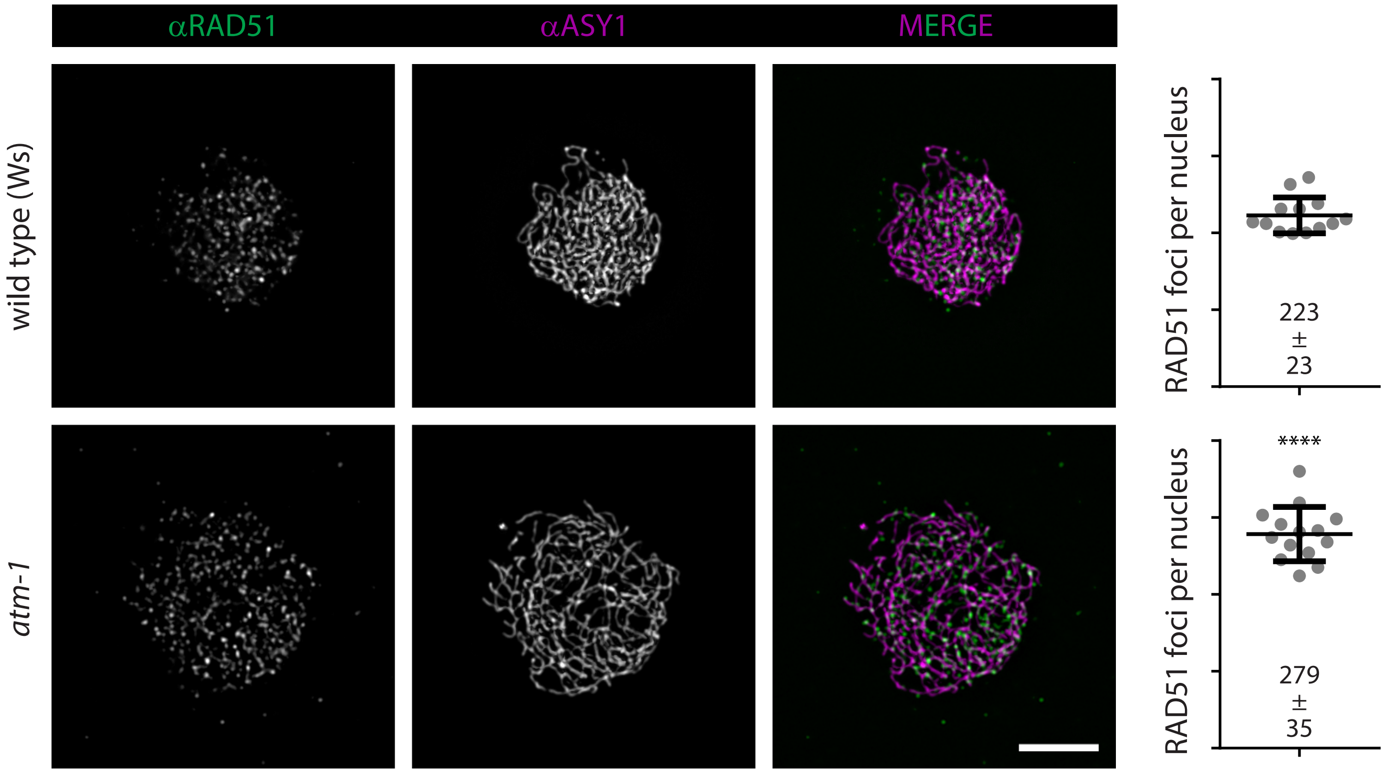

Supplemental Figure 3 . RAD51 foci numbers are elevated in *atm-1*. Meiocytes from the wild-type Wassilewskija had 223 ± 23 RAD51 foci (n = 13 nuclei), against 279 ± 35 RAD51 foci in the *atm-1* mutant (n = 14 nuclei), which represents a significant increase ($p < 0.0001$). Bar: 5 µm. Error bars represent standard deviations.

Supports Figure 2.

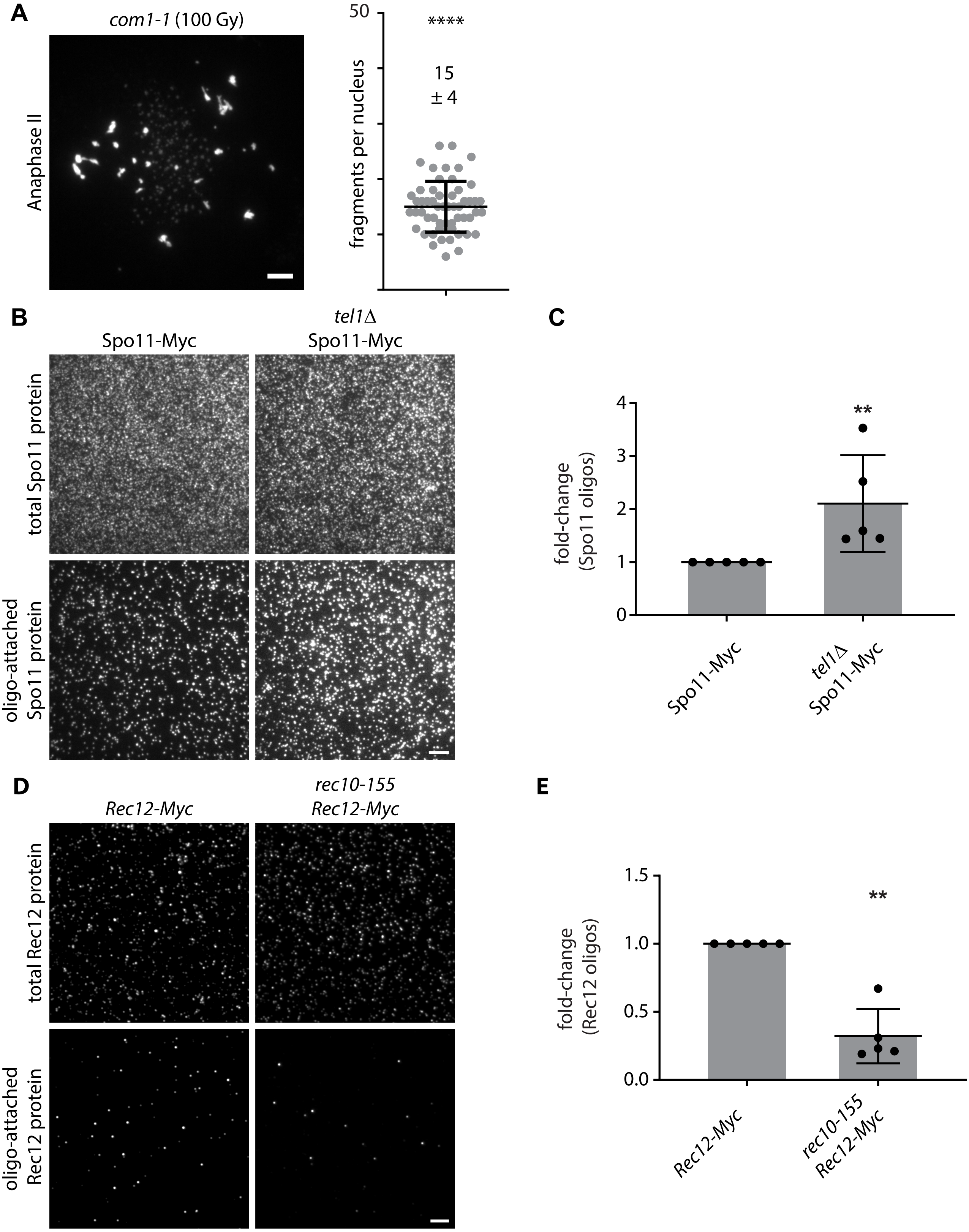

Supplemental Figure 4. Quantification of double-strand break (DSB) levels.

(A) DNA fragmentation as an indirect readout for DSB levels. The number of chromosome fragments significantly increases from 12 (± 6; n = 94 nuclei) in untreated *com1-1* nuclei, to 15 (± 4; n = 55 nuclei; p < 0.0001) upon ionizing radiation treatment.

(B) Tel1 limits DSB formation in *S. cerevisiae*. SIM-TIRF microscopy was applied to image single molecules of total Spo11-Myc protein and oligo-attached Spo11-Myc in wild-type and *tel1Δ* mutant backgrounds.

(C) After normalization to total protein levels, a 2.11-fold increase of oligo-attached Spo11 was found in *tel1 Δ* mutants relative to the wild type (p = 0.0079; five biological replicates).

(D) DSB formation in *S. pombe* largely depends on Rec10. SIM-TIRF microscopy was applied to image single molecules of total Rec12-Myc protein and oligo-attached Rec12-Myc in wild-type and *rec10-155* mutant backgrounds.

(E) After normalization to total protein levels, a steep decrease of oligo-attached Rec12 was found in *rec10-155* mutants (p = 0.0079; five biological replicates). Bars: 5 µm. Error bars represent standard deviations. Supports Figure 3.

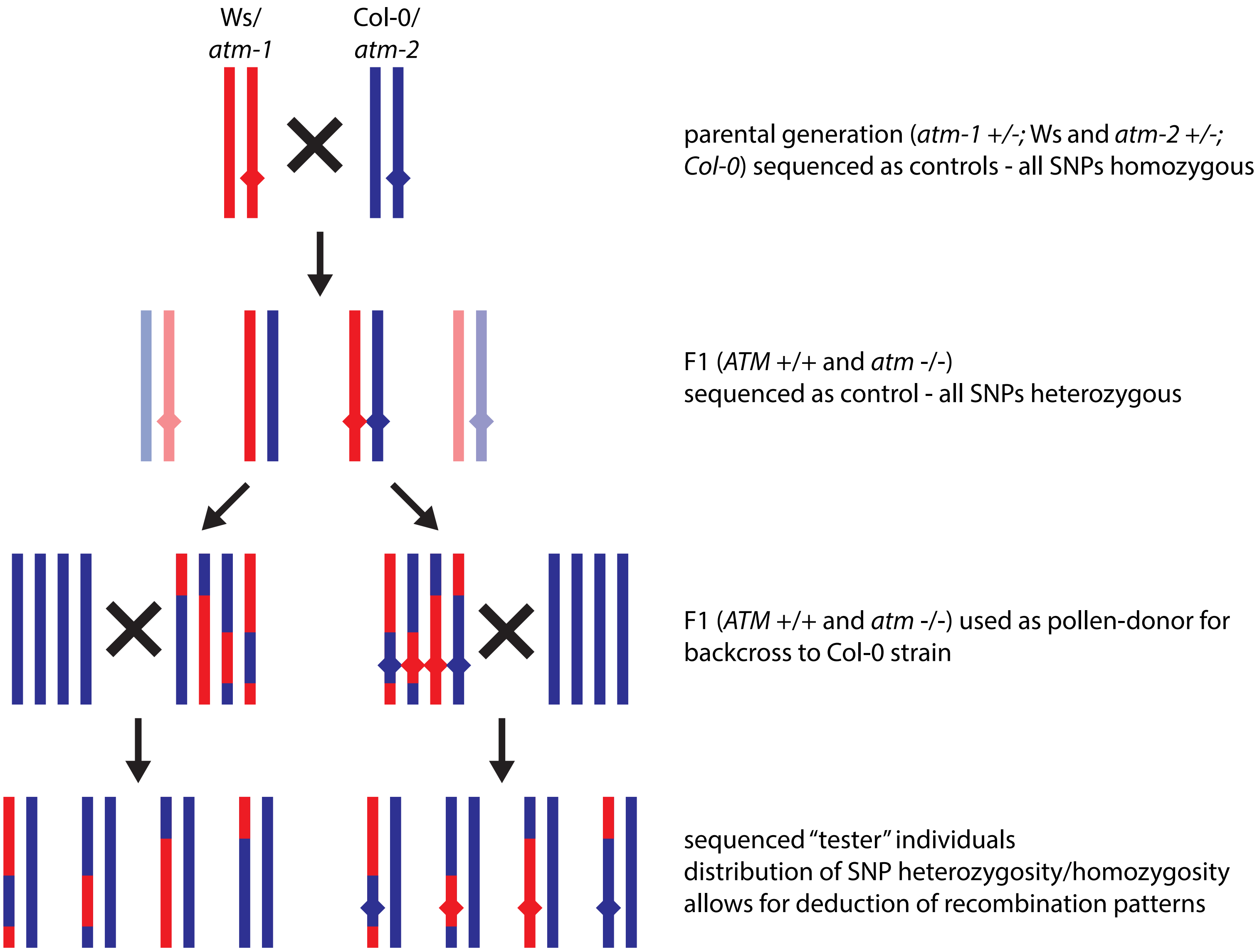

Supplemental Figure 5. Crossing scheme for NGS-based recombination screen.
Supports Figure 5.

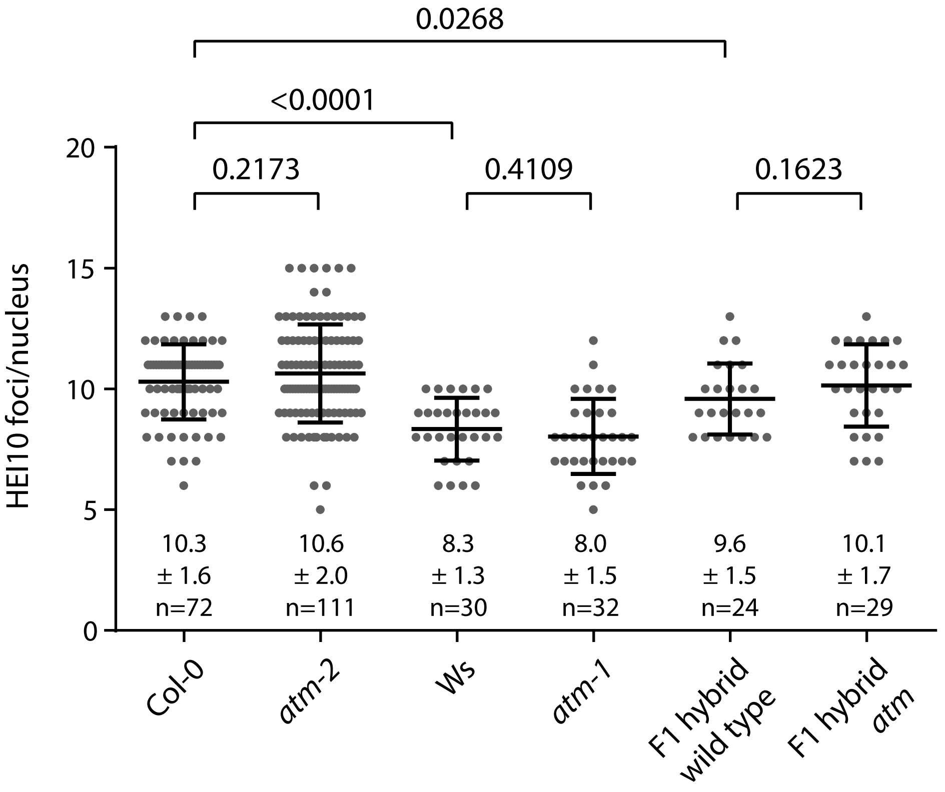

Supplemental Figure 6. The number of class I COs depends on the accession but not on ATM.

Spreads of pollen mother cells were stained for the synaptonemal complex (SC) protein ZYP1 and the E3 ubiquitin ligase HEI10. HEI10 foci were counted. Error bars represent standard deviations.

Supports Figures 5 and 6.

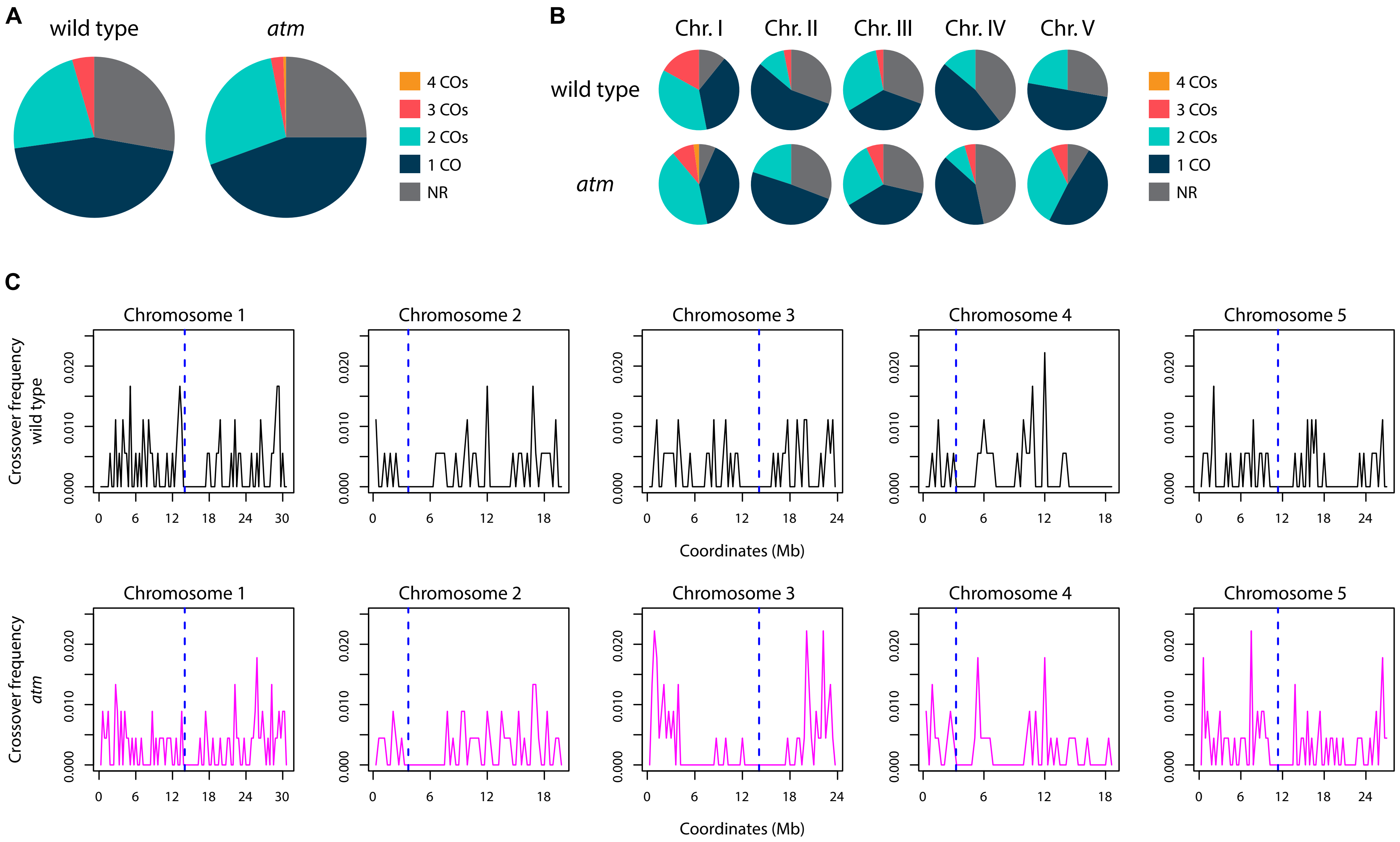

Supplemental Figure 7. Crossover frequency and distribution in wild type and *atm* mutant plants.
(A) Proportion of chromosomes with no (grey), one (dark blue), two (light blue), three (red) or four (orange) crossovers in wild-type and mutant plants.
(B) Same as in **(A)**, per chromosome.
(C) Crossover frequencies and distributions along the five Arabidopsis chromosomes in wild-type (black) and *atm* mutant samples (red). Blue dashed lines indicate centromere positions. The *ATM* gene is located on chromosome 3 and starts at position 17,801,289.
Supports Figure 5.

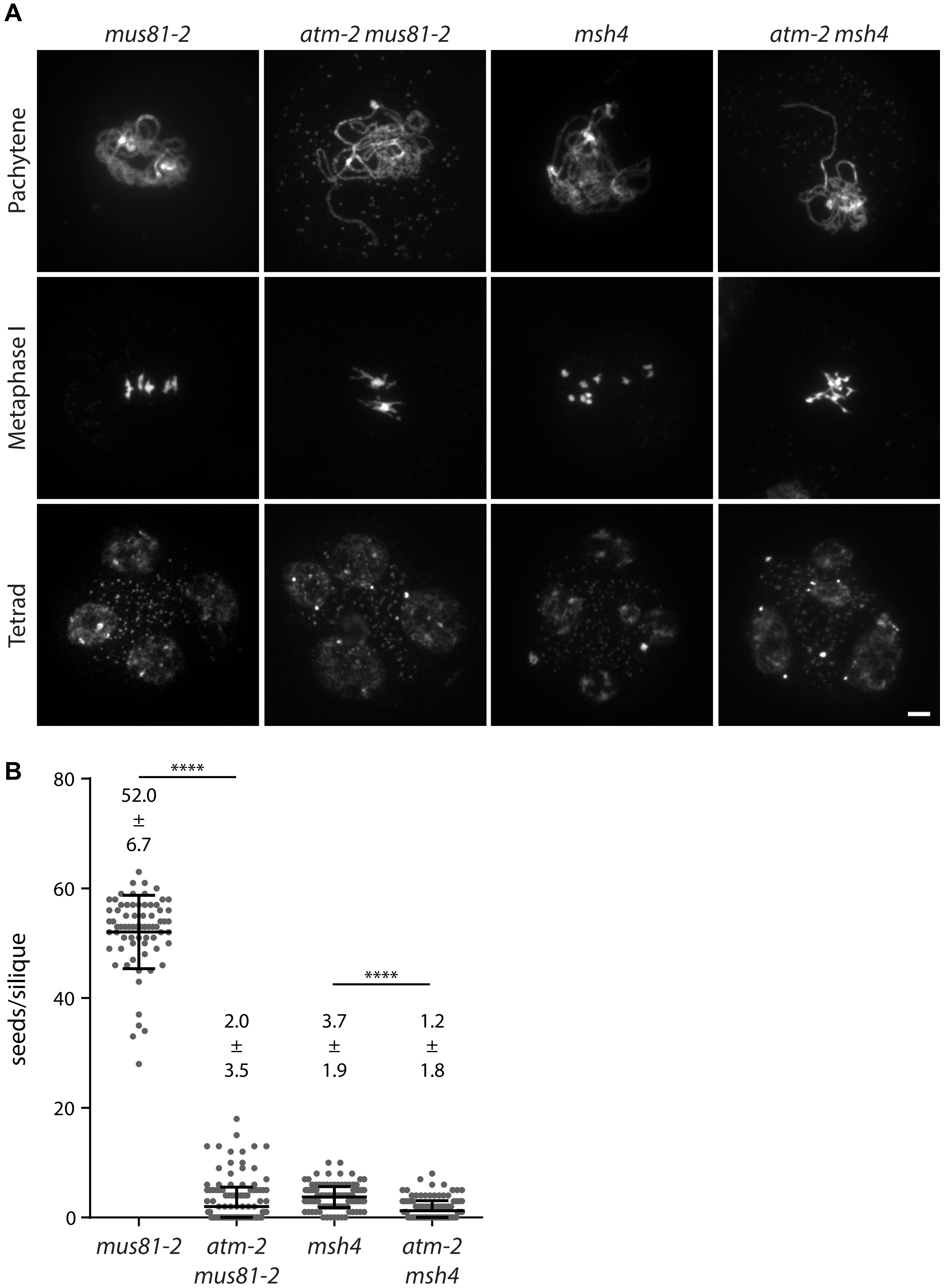

Supplemental Figure 8. Meiotic progression and fertility in *mus81-2, atm-2 mus81-2, msh4* and *atm-2 msh4* mutant plants.

(A) Meiosis in all mentioned mutants appears normal up to pachytene stage, where complete synapsis is observed. Metaphase I chromatin appears entangled and pronounced fragmentation is observed at later stages when *ATM* is mutated. Bar = 5 µm.

(B) *Mus81-2* mutants produce 52.0 ± 6.7 (n = 70 siliques), *atm-2 mus81-2* double mutants 2.0 ± 3.5 (n = 167 siliques; p < 0.0001), *msh4* plants 3.7 ± 1.9 (n = 161) and *atm-2 msh4* plants 1.2 ± 1.8 (n = 126 siliques; p < 0.0001) seeds per silique. Error bars represent standard deviations. Supports Figure 6.

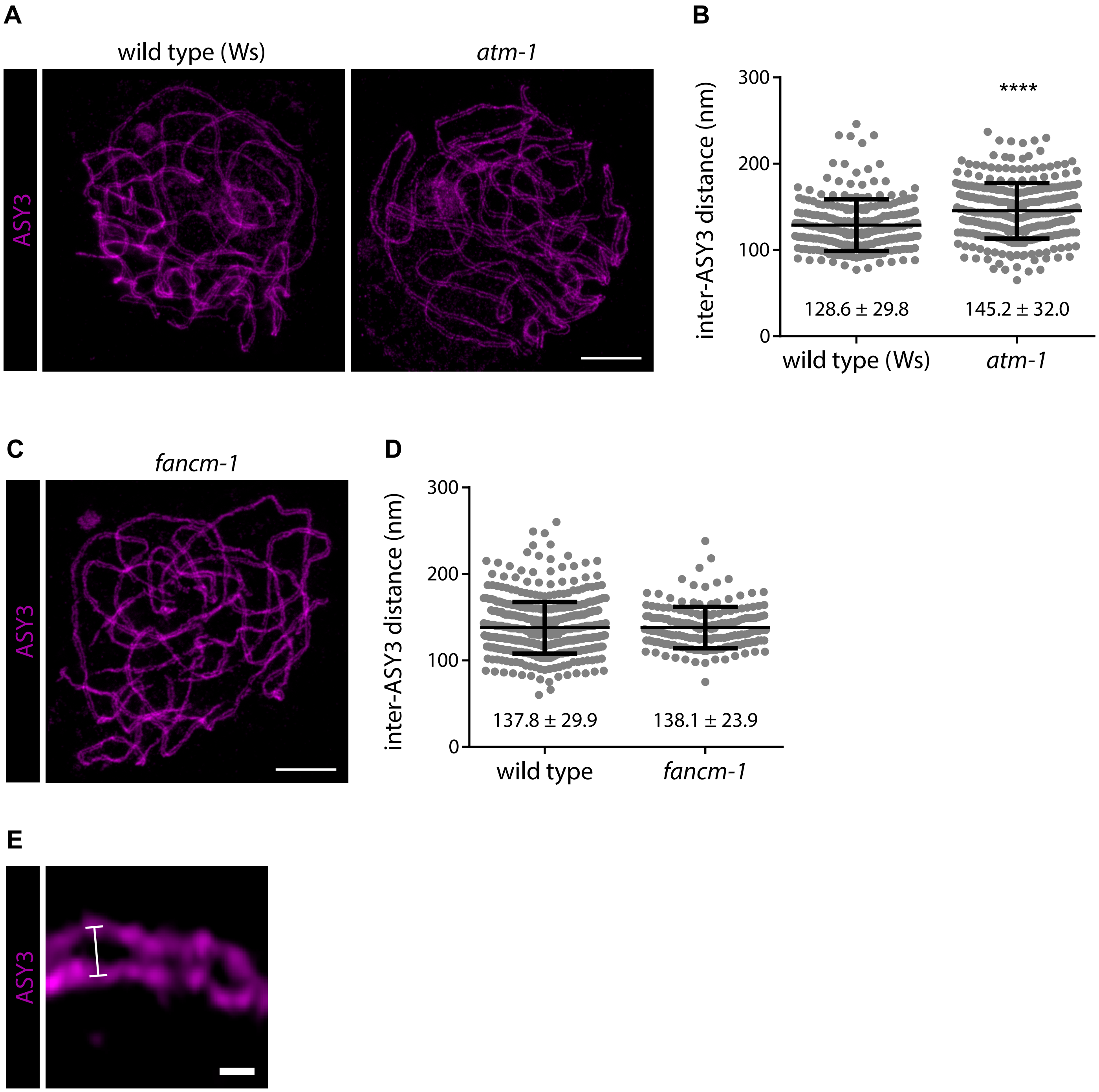

Supplemental Figure 9. The distance between the lateral elements along synapsed chromosomes increases in *atm-1* mutant plants but is not affected by the *fancm-1* mutation.

(A) Pachytene nuclei were stained for the lateral-element-like protein ASY3. Bar: 2 µm.

(B) Inter-lateral-element distances were measured between the centers of ASY3-labeled stretches along pachytene chromosomes and found to increase in the absence of functional ATM (226 measurements in six wild-type (Ws) nuclei; 312 measurements in eight mutant nuclei; $p < 0.0001$).

(C) Spreads of *fancm-1* pachytene nuclei were stained for ASY3. Bar: 2 µm.

(D) Inter-lateral-element distances were measured and found to be unaffected by the increased number of class II COs in *fancm-1* mutants (488 measurements in 12 wild-type nuclei; 180 measurements in nine mutant nuclei; $p = 0.5882$) Bar 2 µm.

(E) Unprocessed STED images were used to measure the distance between the centers of ASY3-labelled chromosome axes in pachytene nuclei. Only chromosomal stretches with the SC in assumed frontal view (local maximum distance between ASY3 stretches) were considered. Bar 100 nm. Error bars represent standard deviations.

Supports Figure 8.

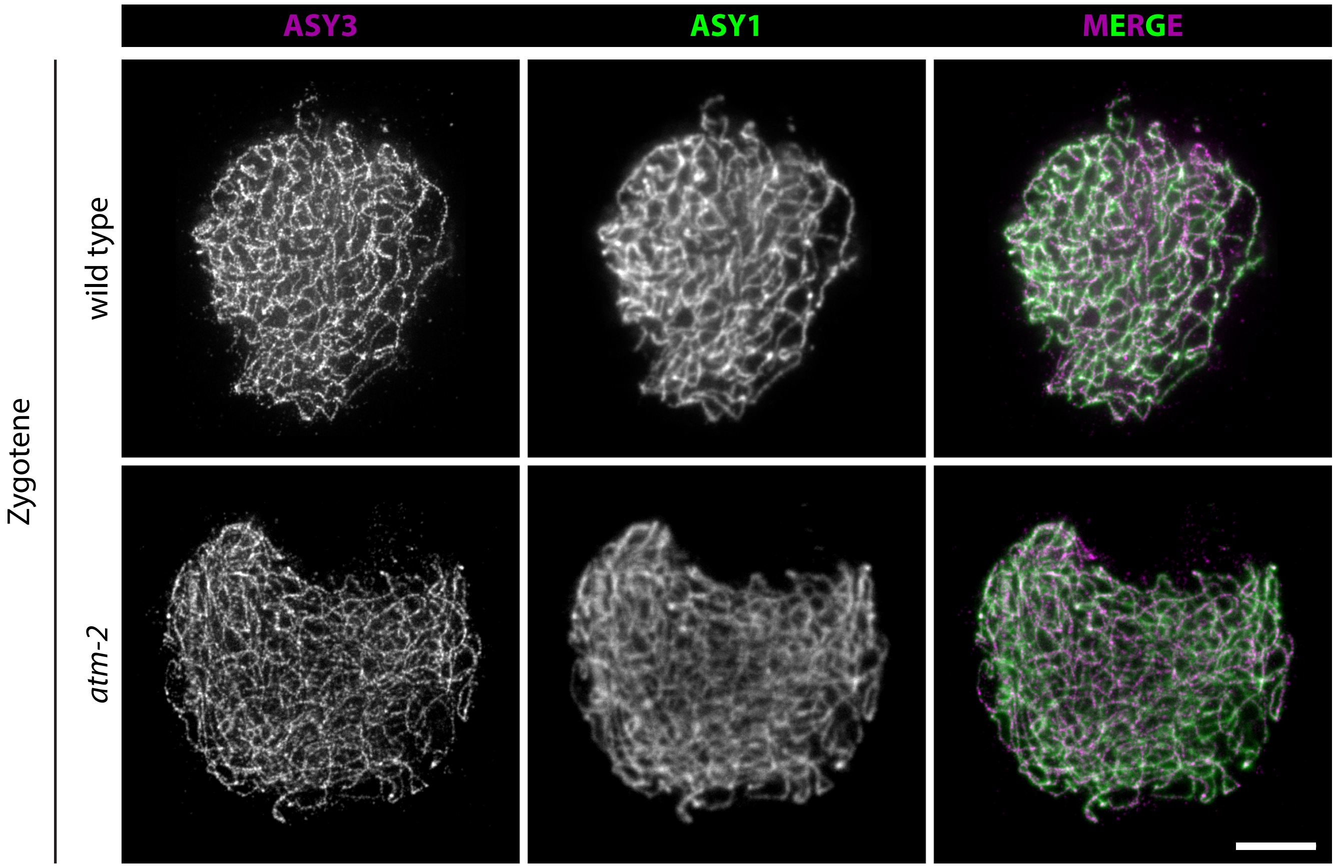

Supplemental Figure 10. Axis formation is normal in *atm-2* mutant plants.
Wild-type and mutant pollen mother cells were stained for the axial element proteins ASY1 (green) and ASY3 (magenta) and analyzed by STED nanoscopy. Bar: 2 µm.
Supports Figure 8.

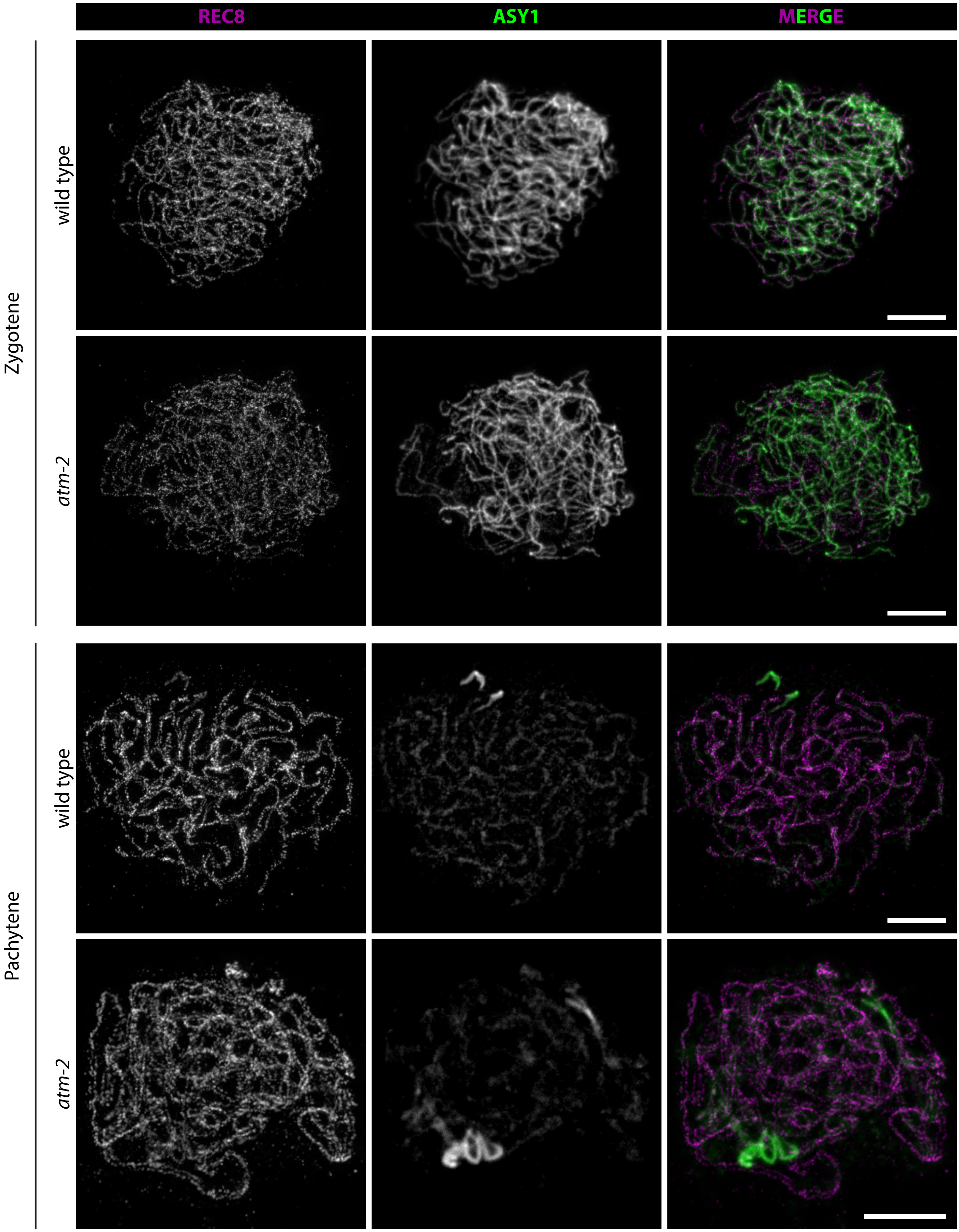

Supplemental Figure 11. Cohesin deposition is normal in *atm-2* mutant plants.
Wild-type and mutant pollen mother cells were stained for the axial element protein ASY1 (green) and the meiotic cohesin subunit REC8 (magenta) and analyzed by STED nanoscopy. Meiotic stages are indicated. Bars: 2 µm. Supports Figure 8.

Supplemental Table 1. SIM-TIRF Data Overview.

Genotype	Spo11/Rec12 Proteins	Spo11/Rec12 Oligos	Fold-change Oligos	Images (n)
S.c. SK1; *Spo11-Myc18*	12,072,940	26,569	1.00	9
	22,851,900	57,290	1.00	20
	17,679,060	8,781	1.00	20
	23,384,400	31,440	1.00	19
	23,058,200	34,573	1.00	20
S.c. SK1; *tel1 ; Spo11-Myc18*	9,457,800	29,960	1.44	9
	19,612,980	71,514	1.45	20
	9,118,800	11,393	2.52	20
	11,079,000	52,594	3.53	19
	15,995,000	38,032	1.59	20
S.p.; Rec12-Myc13	698,250	1,046	1.00	10
	3,362,596	6,391	1.00	10
	2,202,165	430	1.00	15
	1,945,460	5,262	1.00	10
	2,802,800	3,082	1.00	10
S.p.; rec10-155; Rec12-Myc13	673,759	675	0.67	10
	4,747,699	2,054	0.23	10
	5,794,105	355	0.31	15
	3,364,680	1,945	0.21	10
	3,111,900	644	0.19	10
A.th. SPO11-1-Myc18	1,014,250	1,130	1.00	11
	8,160,000	5,508	1.00	18
	31,611,600	1,263	1.00	20
	6,943,800	1,062	1.00	20
A.th. atm-2 ; SPO11-1-Myc18	828,010	1,434	1.55	11
	6,612,070	7,723	1.73	18
	32,284,650	3,333	2.58	20
	8,541,675	2,103	1.61	20

Supplemental Table 2. Raw data of tetrad analysis.

Tetrad numbers are given by interval, genotype and recombination pattern (classification as in Figure 3B). Wild-type data obtained in parallel and also part of (Kurzbauer et al., 2018).

Interval	Genotype	a	b	c	d	e	f	g	h	i	j	k	l	SUM
I2ab	wild type	3,872	261	400	3	1	0	1	1	3	0	0	0	4,542
	atm-2	426	44	50	3	3	1	3	4	1	0	0	0	535
I5cd	wild type	3,609	504	538	7	10	6	3	3	2	0	0	0	4,682
	atm-2	505	78	90	7	2	2	2	1	5	1	3	0	696

Supplemental Table 3. Raw data of I5d single pollen grain analysis.

Genotype	nonrecombinant	recombinant
wild type	4,111	271
atm-2	2,432	291
msh4	3,971	67
atm-2 msh4	1,567	118
wild type	11,316	662
atm-2	4,836	569
mus81-2	8,884	361
atm-2 mus81-2	3,851	279

Supplemental Table 4. Yeast strains used in this study.

Strain	Genotype
S.c. wild type ; untagged	*SK1 MATa/alpha, ho::LYS2, ura3, leu2::hisG, trp1::hisG, his3::hisG, lys2*
S.c. wild type ; Spo11-Myc18	*MATa/alpha, ho::LYS2, ura3, leu2::hisG, trp1::hisG, SPO11-Myc18::TRP* (Prieler et al., 2005)
S.c. tel1Δ ; Spo11-Myc18	*SK1, MATa/alpha, ho::LYS2, ura3, leu2::hisG, trp1::hisG, his4B, tel1::KanMX, SPO11-Myc18::TRP1*
S.p. Rec12-Myc13	*h+ pat1-114 rec12+::13xMyc*
S.p. rec10-155 Rec12-Myc13	*h+ pat1-114 leu1-32 rec10-155::LEU2 rec12+::13xMyc*

Supplemental Table 5. Oligonucleotides used in this study.

Name	Sequence (5′ -> 3′)
ATM104	TGG CAG CCG AGT ATT TTT CAA CTT T
ATM123	ATG AAC TTG GAA GGG TTA CAA GA
ATM-F1	GCT TCA AGG TTG GGC AGT TCC
ATM-R1	GCA AAC AGC ATA ACA AAA CAC TTC C
c-myc_new_dn	ATC CAT GGC TCC CGG GTC CGG TTC TGC TGC TAG TGG
Com1dn	TGT TGC AGG TTA AGG GTT TGG
Com1up	CAT TTC GGA TTC AAA CCC GAT GTT C
FANCM_CAPS_F	ACA ATA TAT GTT TCG TGC AGG TAA GAC ATT GGA AG
FANCM_CAPS_R	CAC CAA TAG ATG TTG CGA CAA T
GABI-1	CCC ATT TGG ACG TGA ATG TAG ACA C
gAtSPOdn	AAA ACT GCA GAA TGT CGT CGG CGG ACA ACA CG
gAtSPO_STOP_dn	TCA ACC CGG GTG AAG ATC TGC TTC ATA CGA GAT AAC TAG TCT CC
gAtSPOup	CGC GGA TCC GCT GAA GCT GAA GTT GCC ACG
gAtSPO_STOP up	CGC GGA TCC TCA **CCC GGG** AGG AGA GCT TAC TTC ACG ACG AAT C
Lig4-8	GTG ATT TGA AAC TAG TCT GTG
Lig4-9	CAG CAA ACC GAT TCA GAG ATG
MSH4_F1	CGC ATA TGG CGC TTG GTT TAG ACA CTT AC
MSH4EXP_R1	GCG TTG TGG AAT GGA TCA ATG
Mus81_LP	TGG TGA AAT CTA GCA ACC CAG
Mus81_RP	AAT TTT CCA CAA ACC CTT TGG
pNOSdn	GGG TTT CTG GAG TTT AAT GAG CT
SALK LBa1	TGG TTC ACG TAG TGG GCC ATC G
SALK LBc1	TGG ACC GCT TGC TGC AAC TCT
SpoSeq5UTR2up	CAA CAC TGA TAA CAT TTA AAT TGC
*spo11-1*_allele_new	GGT TTC GTC TAC CAT CAT CCC AGG C
*spo11-1*_pin_up	CAC GGT CCA CAA TGG ATT GTG CTG
*spo11-2*_down	GCT CGT GGA AGA TCG TGT GTT C
*spo11-2*_up	CCT GCA TAG GAA AGT GGA GAT TAG GAC
TAG3	CTG ATA CCA GAC GTT GCC CGC ATA A
Tn5ME-A	TCG TCG GCA GCG TCA GAT GTG TAT AAG AGA CAG
Tn5ME-B	GTC TCG TGG GCT CGG AGA TGT GTA TAA GAG ACA G
Tn5Merev	[phos]CTG TCT CTT ATA CAC ATC T

Supplemental Table 6. SNP distribution along the five Arabidopsis chromosomes.

Chromosome	Number of SNPs	Proportion of SNPs per chromosome
1	116,574	25.48%
2	73,467	16.05%
3	95,602	20.89%
4	61,736	13.49%
5	110,212	24.09%
Total	457,591	100.00%
