# Supplementary material for: ATM controls meiotic DNA double-strand break formation and recombination and affects synaptonemal complex organization in plants
Source: Plant Cell. 2021 Feb 5;33(5):1633–56. doi: 10.1093/plcell/koab045 (PMC8254504; doi:10.1093/plcell/koab045)
Supplement: koab045_Supplementary_Data [file koab045_supplementary_data.zip › tpc.00768.2020-s06.pdf]

ATM Controls Meiotic DNA Double-Strand Break Formation and Recombination and Affects Synaptonemal Complex Organization in Plants

Marie-Therese Kurzbauer, Michael Peter Janisiw, Luis F. Paulin, Ignacio Prusén Mota, Konstantin Tomanov, Ondrej Krsicka, Arndt von Haeseler, Veit Schubert and Peter Schlögelhofer

Corresponding author: Peter Schlögelhofer peter.schloegelhofer@univie.ac.at

Review timeline:

TPC2020-RA-00159	Submission received:	Feb. 26, 2020
	1st Decision:	Mar. 30, 2020 *decline, might reconsider*
**TPC2020-RA-00768D**	Submission received:	Oct. 05, 2020
	1st Decision:	Oct. 25, 2020 *request revisions*
**TPC2020-RA-00768DR1**	1st Revision received:	Dec. 18, 2020
	2nd Decision:	Dec. 23, 2020 *accept with minor revisions*
**TPC2020-RA-00768DR2**	2nd Revision received:	Jan. 19, 2021
	3rd Decision:	Jan. 19, 2021 *accept*
	Final acceptance:	Jan. 29, 2021

REPORT: (The report shows the major requests for revision and author responses. Minor comments for revision and miscellaneous correspondence are not included. The original format may not be reflected in this compilation, but the reviewer comments and author responses are not edited, except to correct minor typographical or spelling errors that could be a source of ambiguity.)

TPC2020-RA-00159 1st Editorial decision – *decline, might reconsider* Mar. 30, 2020

Thank you for choosing to send your manuscript entitled "ATM Controls Meiotic DNA Double-Strand Break Formation and Recombination in Plants" for consideration at The Plant Cell. Your submission has been evaluated by members of the editorial board as well as expert reviewers in your field, and we regret to inform you that we are not able to recommend publication of this manuscript. We have not made this decision lightly. We have had input from multiple scientists, and we have solicited post-review comments as well. Our present policy is to offer streamlined decisions and to not advise on the direction of the work by requesting extensive modifications or substantial additional experiments.

As you will see, the three referees thought that the work is of high quality and interesting, increasing our understanding of the role of ATM in DBS regulation during meiosis and of synaptonemal complex structure. The work also contains novel technical aspects. However, the reviewers had many concerns: they requested additional controls to substantiate several points (see such requests by reviewers 1 and 2). Reviewer 2 was concerned by the reliance on *atm-2*, which lacks in depth characterization, and thought that there is a need for a second allele. We should remind you of this text from our instructions to authors document, as it may be relevant, "Generally, characterization of multiple, independent alleles is necessary to establish that a mutation (such as a T-DNA insertion or chemically induced mutation) is responsible for an observed phenotype, as opposed to an undetected, linked mutation. Complementation tests via transformation can be valuable, although their interpretation may be limited by the possibility of redundancy, and thus statements of proof should be limited to cases in which multiple independent alleles have been characterized."

There was also some concern on the lack of robust/reliable statistics when measuring some of the effects. Moreover, while the referees did appreciate the novel insight provided by your work, they also felt that novelty is limited as

similar results were reported in other species. Addressing all the reviewers comments would involve extensive experimental work.

That said, we would be willing to re-consider a new manuscript that fully addressed the concerns raised during this review process. It would be also important to convince the editors and reviewers that novelty is significant. If you decide to resubmit to The Plant Cell, it will be evaluated as a new submission subject to full assessment by the editorial board, and if sent for external review, a new set of reviewers is likely to be chosen. Reviewers will be asked to assess your work as a new manuscript (i.e. are the claims fully supported by the data and, do the results presented move the field forward?), and not only whether previous reviewer comments have been addressed.

TPC2020-RA-00768D	Submission received	Oct. 05, 2020

--- Reviewer comments (with **Authors response in bold**):

Dear Editors,

We are very grateful for the reviewers' comments and addressed all points raised, significantly improving our study. Please find a point-by-point response to the concerns raised below (remarks are kept in *italics* and our response in regular letters).

Overview of added experiments and implemented changes:
New experiments:

RAD51 foci in *atm-1* and the corresponding wild type (Ws)
FTL recombination screen on *atm-2 msh4* double mutants
measurement of chromatin loop size using STED nanoscopy
estimation of loop density
measurement of inter-axis distances in *atm-1*
seed counts repeated and extended (new genotypes: *atm-1*, Ws, *atm-2 mus81-2*, *atm-2 msh4*) spreads of *atm-2 mus81-2*, *atm-2 msh4* double mutants included

Changes:

the title was extended
several sections were re-written, see below for details
parts of the results were moved to introduction
figures were rearranged to increase clarity
discussion was extended
supplementary tables with raw data were added

Reviewer #1 (Comments for the Author):
In this manuscript, authors Marie-Therese Kurzbauer et al. further explore the role of ATM in meiotic recombination in Arabidopsis thaliana. It was previously known than ATM was required for proper DSB repair at meiosis in Arabidopsis, and that ATM was involved in the regulation in both DSB formation and repair in other model organisms. The new results include the observation of DMC1 and RAD51 foci in the atm mutant, an analysis of the number of DSBs in the mutant, an observed increase of recombination in atm with FTLs and genome wide sequencing, and the observation that the SC is longer and wider in atm than in wild type. Altogether this works adds some interesting information on ATM, without generating a quantum leap for our understanding of its function at meiosis.

I have some comments and suggestions:

Major comments:

Point 1. *The current version of the manuscript does not always make clear what is novel and what was previously published. This should be corrected.*

RESPONSE: We rearranged several parts of the manuscript and improved clarity. The first part of the results section is now included in the introduction and the needed references were added.

Point 2. *L112-114. It should be mentioned here that a role of ATM in meiotic DSB repair was demonstrated before in Arabidopsis (Garcia et al 2003, Culligan and Britt 2008).*

RESPONSE: We did mention that and precisely stated: "The Arabidopsis ATM protein has been proven to play a role in DNA damage sensing but has not been implicated in regulating DSB or CO formation, to date". The sentence was changed and new references added.

Point 3. *L126-134. This is published work and should be moved to the introduction*

RESPONSE: We agree and moved the part to the introduction.

Point 4. *L134-138. This dataset has been published previously by the same group. It should be made clearer.*

RESPONSE: We did give reference to our earlier published data. In order to avoid any ambiguity and to properly include data on a second mutant allele, *atm-1*, we performed a new seed count. All values were updated and are found in Suppl. Figure 1.

Point 5. *L140-142. The spo11-1 atm double mutant was described previously (Culligan and Britt 2008).*

RESPONSE: Yes, we agree, but it should be noted that the previously analyzed double mutant was *spo11-1-1 atm-1,* with the *spo11-1-1* allele not being a complete loss-of-function allele (but the only one available then). We repeated the analysis with the *spo11-2-3* allele, where DSB formation is completely abrogated. We extended our writing for more clarity and included the reference.

Point 6. *L145-146. This means 2/49 in atm versus 4/98 in atm lig4. This is not enough to draw a robust conclusion. The numbers should be increased or the result deleted. In addition, supporting pictures should be shown (possibly in sup data).*

RESPONSE: We think that the number of analyzed pictures (53 for *atm* and 98 for *atm lig4*) represents a solid base for our statement, especially when compared to other cytological studies. Please note that supporting pictures were provided and are shown in Suppl. Figure 2.

Point 7. *L159-210. This is the main novelty of the manuscript, with an attempt to estimate the number of DSBs in atm. The author developed a novel technique for that purpose, which is interesting. However, a negative control is missing. Using a SPO11 catalytic mutant (Y135F) would be a good option. Additionally, the author could also control that no increase in DSBs is observed in com1.*

RESPONSE: We agree with the reviewer that a "catalytic dead" SPO11-1 mutant (in case of Arabidopsis SPO11-1 it would be Y103F) would be an ideal control. Unfortunately, we do not have such a control at hand. We would like to highlight the "proof-of-principle" experiment performed in *S. cerevisiae* (Suppl. Figure 4) and also the supportive independent experiment performed with counting fragments in *com1-1* and *com1-1 atm-2* mutants, corroborating the single-molecule approach. In *com1-1* mutants the release of SPO11-1 from meiotic chromatin is strongly delayed (Uanschou et al., 2007) but not abolished. Given the fact that in plants we cannot stage individual meiocytes for these kinds of biochemical experiments we do not anticipate that *com1-1* mutants represent a suitable negative control.

Point 8. *L222-235. The wild type data used here were the controls of a previous experiment published by the same team (as a control for the fancd2 mutant. Kurzbauer et al 2018). Were all the plants grown together? If not, I would suggest to include some wild type control grown together with the atm mutants to account for environmental effects on recombination.*

RESPONSE: All compared plants were grown at the same time, side-by-side and under rigorous environmental control. We did, however, repeat the seed per silique count for the current version of this manuscript to avoid ambiguity and also included information on *atm-1*.

Point 9. *L304. As the author cherry-picked some chromosomes (with larger differences), the statistical test is no longer appropriate. The difference is thus not statistically significant (but the number of samples is rather low). The sentence in the abstract "genome-wide recombination screens showed that ATM restricts the number of interference-insensitive crossovers" should be also edited.*

RESPONSE: Here we strongly object. We do not "cherry-pick". We mention the results in an unbiased manner for the entire genome and then group those three chromosomes that do not contain a NOR and those two that contain a NOR. NOR-containing chromosomes have been shown in the past to differ in their behavior compared to other chromosomes in mutants that affect homologous recombination (Lam et al., 2005; Sims et al., 2019). We re-named the groups of chromosomes in "short (NOR-bearing) chromosomes" to avoid the impression of arbitrary grouping.

Minor comments

Point 10. *L151. "Formed" should be replace by "observed". As the author mentioned later in the manuscript, it could be a difference in the turnover.*

RESPONSE: Done.

Point 11. *L167. I would avoid the jargon PMC and use the more generic "male meiocytes"*

RESPONSE: We avoid the use of the abbreviation PMC in the current version of the manuscript.

Point 12. *L229-230. Raw data, plant per plant, should be provided as sup data.*

RESPONSE: Raw data are now given in Suppl. Table 2.

Point 13. *L256. I do not see any mention of atm in Jahns et al 2014.*

RESPONSE: The article is cited because of the description of HEI10 foci clustering. The sentence was rewritten for clarity.

Point 14. *L288. All the sequencing data should be deposited in a public database.*

RESPONSE: All sequencing data has been deposited in a public database. Please refer to the Material and Methods section: "Data availability: All sequences obtained by deep sequencing have been uploaded to the NCBI public database (project PRJNA555773)."

Point 15. *L351. "precise map of ATM function" is an overstatement*

RESPONSE: Changed.

Point 16. *L397. It could be also a pure line vs hybrid difference.*

RESPONSE: We think this is rather unlikely, as HEI10 foci numbers are unaffected. Furthermore, the other experiment evaluating recombination (FTL assays) was performed in an isogenic background and did also show a recombination increase.

Point 17. *L403. This is also true for all the other experiments.*

RESPONSE: We are not sure to which other experiments the reviewer is referring to.

Point 18. *Figure 4B. I suspect a mistake in the drawings as CO figures in a and d are identical (d is likely a double CO)*

RESPONSE: This was a mistake and was corrected (now Figure 3B).

Reviewer #2 (Comments for the Author):

This manuscript by Kurzbauer et al. reports on the previously well-studied ATM, a crucial protein kinase during meiosis in Arabidopsis. ATM is a huge protein with almost 4000 aa in length and plays important roles in somatic and meiotic recombination. In this study, the authors conducted multiple approaches including new technology to re-investigate the function of ATM during meiosis, with many focus on atm-2 allele. In comparison to the previously-identified roles

of ATM, this paper also provided several lines of evidence that ATM participates in multiple processes during meiosis, including negative regulation of DSB formation, limiting meiotic MUS81-dependent COs and maintenance of synaptonemal complex length and width.
Overall, although ATM is well known in meiosis, the paper has indeed identified the new function of ATM in meiosis beyond what we have known from previous publications. The paper is a great contribution to this field and is also helpful for people to comprehensively understand the role of ATM in meiosis. Moreover, the paper apparently used new approaches such as single-molecule analyses to generate the data to strongly support their conclusion. However, I have several concerns or comments for the authors' consideration to further improve the manuscript, which should be addressed.

Major concerns:
***Point 1**. As descried above, ATM is a huge protein and has several different mutant alleles, which gave varied phenotypic differences from previous reports. The present paper only used one allele of atm-2, but I did not see the description of atm-2 in detail as compered compared with other alleles throughout the manuscript including methods. Why did the author focus on this allele? This is the critical point what I worried about the central conclusion drawn by the authors. Is atm-2 null allele? Or there is still expressed truncate ATM protein in atm-2. This should be examined. Alternatively, I strongly recommend the authors to study another independent allele. It is also possible to obtain inconsistent phenotypes between different alleles, since this paper used new technology, which I believe can make the data comparable and conclusion stronger.*

RESPONSE: Certainly, we agree with the reviewer and the editor that new mutant alleles need thorough characterization and mutant phenotypes need to be confirmed by additional alleles. Yet, the *atm-2* mutant allele (and the *atm-1* allele) employed in our study is not a new allele and has been thoroughly characterized in previous studies (Garcia et al., 2003; Friesner et al., 2005; Vespa et al., 2005; Culligan et al., 2006; Ricaud et al., 2007; Vespa et al., 2007; Adachi et al., 2011; Amiard et al., 2011; Bohmdorfer et al., 2011; Hisanaga et al., 2013; Yoshiyama et al., 2013; Liu et al., 2015; Roitinger et al., 2015; Wang et al., 2016; Waterworth et al., 2016; Lorkovic et al., 2017; Su et al., 2017).

While the experiments performed in the publications mentioned above demonstrate that *atm-1* and *atm2* represent null alleles, we did follow the suggestions by the Editor and Reviewer #2 and repeated several key experiments with the independent *atm-1* allele. All results were virtually identical to the ones found with *atm-2* mutants and include: Seed/silique count (Suppl. Figure 1C) RAD51 foci (Suppl. Figure 3) HEI10 foci (Suppl. Figure 6) Inter-axis distance (Suppl. Figure 9)

***Point 2.** Several controls are missed throughout the manuscript. For example, Figure 2 should include WT with or without Gy treatment, and the double mutant with Gy treatment.*

RESPONSE: We agree that we could have explained the experiment better and therefore changed the corresponding text and representation (Figure 2B and Suppl. Figure 4A). The following rationale is behind the experiment shown in Figure 2: *com1-1* mutants show fragmentation and the regular post-DSB processing is blocked (and SPO11 removal severely delayed and most likely mediated by non-specific DNA repair enzymes; (Uanschou et al., 2007)). Therefore, canonical meiotic DSB repair is blocked and DNA fragments can be used as a proxy to evaluate DSB numbers. We wondered if an increase in DSBs could be detected at all in this set-up and therefore irradiated *com1-1* mutants to create additional breaks. Indeed, additional fragments can be observed in irradiated *com1-1* mutants, providing proof that increased DSB activity can be assayed. Therefore, irradiated *com1-1* mutants represent the control experiment. We then moved on to the actual experiment, evaluated DNA fragmentation in *com1-1 atm-2* mutants and found that indeed more DSBs are formed in the absence of functional ATM.

***Point 3.** Lack of all controls in SFigure 2 without WT in (A), WT, lig4-4 and atm-2 lig4-4 in (B) and spo11-2-3 in (C).*

RESPONSE: We considered depicting meiotic stages of the requested controls unnecessary, as meiotic progression has been extensively documented in wild-type, *lig4-4* and *spo11-2-3* meiocytes (Ross et al., 1996; Hartung et al., 2007; Dumont et al., 2011).

Point 4. *Similar cases are also found in other experiments.*

RESPONSE: We would like to know in which other experiments so that we can address any potential shortcoming.

Point 5. *I question about the explanation for the observed chromosome bridges in atm-2 mainly caused by NHEJ pathway.*

RESPONSE: The results are clear-cut: in *atm-2* mutants a small proportion of DSBs is repaired in such a manner that bridges are formed. Bridge formation is reduced when the ligase involved in cNHEJ, LIGASE 4, is not present. Therefore, we reason that the occasional, aberrant DNA repair in *atm-2* mutants that leads to DNA bridges (by connecting two unrelated chromosome arms) largely depends on the activity of the cNHEJ pathway.

Point 6. *In Line 142-146, the sentence described "chromosome bridges were observed in 8% of nuclei between anaphase I and tetrad stages (n = 53), while 2% of bridges are remained in atm-2 lig4-4 double mutants, supporting the function of canonical non-homologous end-joining (cNHEJ) in atm-2". Please explain why the bridged chromosomes during meiosis are thought to be generated by NHEJ.* See above.

For statistical counting the fragments, the paper showed that com1-1 mutant had 12 (plus minus) 6 fragments. I am curious about this data. I really don't understand if all DSBs are unrepaired in com-1, why it only yields about 12 fragments. Rad51 mutant is common used in many studies, it seems that the fragments are more than 12 fragments.

RESPONSE: Please refer to the explanations given above. Additionally, we would like to add, that our counting method is very conservative and only fragments that give a more intense signal than the DAPI signals from the organelles are counted. This means that many of the smaller fragments are ignored and actual DNA fragmentation (DSBs numbers) underestimated. Therefore, fragment numbers do not reflect absolute DSB numbers. Furthermore, we are counting DAPI-stained bodies and then provide the number of fragments as "DAPI-stained bodies minus the anticipated number of chromosomes" (as described in the "Materials and Methods" section).

Point 7. *In addition, treatment with ionizing radiation can significantly increase the number of fragments in com-1, but with dramatic evaluation in the additional absence of ATM. Thus, they draw the conclusion that the increased fragments are likely caused by the increase of DSBs in the double mutant. If this experiment can include another controls of spo11-1, or spo11-1 com1, the conclusion will be more convincing.*

RESPONSE: Please note, the *com1-1* mutant has been irradiated as a control (see also explanations given above). The actual experiment investigated fragment numbers in *com1-1 atm-2* double mutants (as a proxy for DSB activity). These plants were not irradiated. We rewrote the corresponding paragraph in the manuscript for more clarity. Investigating *spo11-1* mutants or *spo11-1 com1-1* mutants, where no DSBs are formed, is not relevant for the given experiment and has been done before (Grelon et al., 2001; Stacey et al., 2006; Hartung et al., 2007; Uanschou et al., 2007).

Point 8. *Please explain why the interference-sensitive COs were unaffected with a lost of interference in atm-2.*

RESPONSE: We rewrote the corresponding paragraph for clarity and removed the "interference" indication in Figure 3 (previous Figure 4) as it was indeed misleading. In brief, our findings underline that in *atm-2* mutants the interference sensitive class I CO pathway is still intact. The change of interference ratios in the FTL data set is explained by additional class II COs.

Point 9. *Data supporting the increased COs in atm-2 in MUS81-dependent manner is weak. The authors did not show the phenotypes of fertility and chromosome morphology in atm-2 mus81 double mutant. It is plausible that the double mutant could be infertility and displays chromosome fragments.*

RESPONSE: We consider the data set regarding the analysis of increase of COs in *atm-2* as very solid. We would be happy to receive sound arguments what is considered "weak" in our approach or experimental implementation to be able to respond to the reviewer. Furthermore, we would like to invite the reviewer to consult our previous publication (Kurzbauer et al., 2018) to read about the phenotype of the *atm-2*

mus81-2 double mutants (as indicated in the text). Indeed, there are chromosome fragments in the double mutants and fertility is severely affected. For clarity, we included chromatin spreads and a seed count of _atm-2 mus81-2_ mutants in Suppl. Figure 8. In case we would erroneously score pollen grains with fragmented chromosomes (within the tested interval), then the anticipated result would be an increase in recombination rates and not, as observed, a decrease.

Point 10. *Based on single interval data estimating CO frequency, the author draw the conclusion that CO frequency shows no significant difference between WT and atm mus81 double mutant and that the additional CO in the atm is MUS81-dependent. If so, I strongly recommend the author to examine the HEI10 foci in the double mutant and also provide the metaphase I and anaphase I chromosome morphology.*

RESPONSE: We followed the reviewer's suggestion, performed HEI10 foci counts in _atm-2 mus81_ double mutants and didn't find a change in HEI10 foci numbers (10.96 +/-2.4 foci in _atm-2 mus81-2_; p = 0.4859, n=26 nuclei). The "chromosome morphology" has been published before (Kurzbauer et al., 2018) and is now also included in Suppl. Figure 8: chromosome fragmentation during meiosis.

We now also include an FTL-based recombination assay of _atm-2 msh4_ double mutants (Figure 5), further supporting our claim that class II CO mediators are responsible for the elevated recombination rates in _atm-2_ mutants, while class I factors are not involved.

Reviewer #3 (Comments for the Author):

This manuscript by Kurzbauer et al. presents their characterization of an Arabidopsis mutant that is defective in the DNA damage sensing ATM kinase. Although the role of ATM in the Arabidopsis DNA damage response was initially published well over a decade ago in a series of papers by Britt and colleagues, this new study is focused on uncovering details of its role during prophase I of meiosis. The authors present evidence that indicates that ATM is important for regulating the number of meiotic DNA double-strand breaks (DSBs). They find that the increased level of DSBs is associated with an increase in the number of genetic crossovers, although ultimately the plants exhibit reduced to due errors in break repair. A further finding is that loss of the protein affects the structure of the synaptonemal complex (SC). The manuscript is clearly written and the experiments carefully conducted using a variety of complementary approaches. The results relating to the effect on DSB and CO formation are clearly presented and discussed. These provide a valuable contribution to our understanding of meiotic control in Arabidopsis and are likely relevant to other plants. However, their broader impact is perhaps a little limited by the fact that largely similar findings have been previously described in budding yeast, Drosophila and mouse. Their observation that loss of ATM leads to an alteration in SC structure is novel but as they are, at this point at least, unable to find any biological impact, its significance, if any is intriguing and remains a mystery.

Specific comments.

Point 1*. Lines 140-145; 360-361. The authors provide evidence to show that in the absence of ATM, a small proportion of broken DNA ends are repaired by non-homologous end-joining involving LIG4. In the discussion they refer to this observation stating that "This indicates some DSBs are not processed in a canonical manner". I assume that they are referring solely to the atm-2 line, please clarify.*

RESPONSE: The paragraph was rewritten to avoid ambiguity.

Point 2. *Lines 152-157 The authors report an increase in the foci numbers of the strand-exchange proteins RAD51 and DMC1 at zygotene, indicative of an increase in DSB formation in atm-2. Based on the data presented the actual number of additional DSBs is difficult to assess. It seems that while the number of RAD51 foci at zygotene is increased by 20% relative to wildtype the number of DMC1 foci increases by 74%. In an earlier study (Plant Cell 24, 2058-2070 2012) they reported that maximum numbers of RAD51 foci are seen during leptotene and are decreasing by zygotene whereas the reverse true for DMC1. Would not this suggest that the increase in DSBs is perhaps more accurately reflected by the number of DMC1 foci when determined in zygotene nuclei. Actually this figure more closely reflects the increased number of SPO11 oligos determined using TIRF.*

RESPONSE: We agree with the reviewer that the increase of DMC1 foci numbers is in line with the observed increase of SPO11-oligo numbers in our TIRF experiment. We are cautious in our interpretation of the

observed recombinase numbers, since we do not know how ATM might influence the timing of DNA processing, recombinase loading and strand invasion.

***Point 3**. Lines 159-210 In this section the authors present two lines of analysis that provide further evidence that loss of ATM results in increased levels of DSBs. While I can see that having carried out the experiments the authors wish to include all the data I am not really sure that the fragmentation analysis is really essential to include in the main text. Determining the number of fragments in chromosome spreads is very difficult, some may be very small and overlaps will be common. In a way I think this detracts from the SIM-TIRF approach they have used. Application of this methodology for determining the levels of SPO11 catalyzed DSBs in plant meiocytes is novel and provides strong evidence to back the immunolocalization analysis.*

RESPONSE: In principle we agree with the reviewer, but we felt that a novel method needs corroboration with an established one. We now shortened and re-wrote the section, moved parts of the "DNA-fragmentation experiment" to the supplemental files (Suppl. Figure 4A) and created a single figure with one strong statement (Figure 2) "ATM limits DSBs".

***Point 4.** Lines 211-310 In the next two inter-related sections the authors provide strong evidence to show that the increase in DSB formation arising through the loss of ATM is accompanied by an increase in crossover formation. They provide evidence for this through use of the FTL assay, chiasma counts and NGS sequencing of a Col-0 x Ws cross. They also include evidence to show that, based on an unchanged number and normal patterning of HEI10 foci, that the number and distribution of Class I interference-sensitive COs is unaffected in the atm-2 mutant. In contrast, when they tested an atm-2 mus81 double mutant they found that the overall CO frequency was reduced back to wild-type levels indicating that the additional COs were dependent on an increased level of Class II noninterfering COs. I think that the authors have done a good job in defining the impact of loss of ATM on CO formation and the source of the additional COs.*

RESPONSE: We now also include an FTL-based recombination assay of *atm-2 msh4* double mutants (Figure 5), further supporting our claim that class II CO mediators are responsible for the elevated recombination rates in *atm-2* mutants, while class I factors are not involved.

***Point 5.** Nevertheless, I do think there are a couple of points to raise. First, I found the organization of this section a little odd in that it jumped back and forth between measuring CO formation and investigating the basis of the additional COs. It seems to me that it might be clearer to report the data relating to the increased COs first and then address the basis of the these, that is are they Class I or Class II in origin.*

RESPONSE: We agree with the reviewer and rewrote/reorganized the paragraphs and figures accordingly.

***Point 6**. Second, In lines 231 to 233 they state that based on the FTL data that loss of ATM results in a loss of CO interference. This statement needs to be modified. The FTL assay can reveal a change in CO patterning (which the authors accurately mention in the discussion), however whether this is due to a change in CO interference through an effect on the designation of Class I COs or through an effect on Class II interference-insensitive COs cannot be discerned. In actual fact as they convincingly demonstrate interference appears unchanged in the mutant and that the additional COs arise through the Class II route. Hence, it would be more accurate to say that the FTL data indicates an effect on CO patterning that might be due to an impact on interference but could also be due to elevated Class II COs. They sort of say this but only after they have stated that interference is lost in atm-2, which is clearly not the case.*

RESPONSE: We agree with the reviewer and rewrote the paragraph accordingly and changed the figure to avoid a wrong impression on interference ratio changes (see response to Reviewer 2 above).

***Point 7.** Lines 311-327 The authors present the impact of loss of ATM on axis length at pachytene. This analysis is based on work which reveals a positive correlation between axis length and CO frequency. It is important to note, that as far as I am aware, this correlation is based on the analysis Class I CO frequency, detected as HEI10 or MLH1 foci and axis length. How Class II COs fit into this is unknown and as they are rare and their distribution stochastic, difficult to assess.*

RESPONSE: It is true that HEI10 and MLH1 foci were mostly used for these analyses. Nevertheless, (Giraut et al., 2011) analyzed SNPs in a Col/Ler cross, taking all COs into account. We extended the writing in the discussion to specifically address this point.

Point 8. *The authors report an increase in axis length at pachytene in atm-2. What is most striking about their data is that most nuclei they analysed (14/19 for atm-2; 13/15 for wild-type) had essentially the same axis length but the remaining nuclei in the mutant showed substantial variation sufficient to influence the overall data. Thus while agree there appears to be something going on in relation to axis length in the mutant, based on the evidence presented here it is not clear what that might be. For example, the axis length through prophase I including pachytene is not constant hence if there is a delay in prophase I progression atm-2, say due to the additional number of DSBs, then it may be that the nuclei analysed are not at the same stage. An EdU/BrdU time course would be needed to answer this and/or using dual immunolocalization with ZYP1/HEI10 which can be used to identify cells at late pachytene.*

RESPONSE: We agree with the reviewer that the changes in overall SC length are due to the larger variation measured in *atm-2* mutants. We cannot rule out that an overall mis-coordination of meiotic progression skews our measurements. We are certain that we measure at the correct time, using the axis protein ASY1 an indicator of synapsis progression (full pachytene reached with only two prominent regions in the rDNA stained; (Sims et al., 2019)). We now added a further experiment that corroborates our observations: we find that chromatin loops are in general shorter in *atm* mutant plants, which is consistent with a longer SC.

Point 9. *It is also possible given that Class II CO distribution is stochastic, earlier studies indicated that although they average 1.5 per wild-type cell, the actual range is 0-5, that the cells with longer SCs correspond to those with more Class II COs. It would be interesting to evaluate the Class II CO distribution in an atm-2 zmm mutant.*

RESPONSE: We agree with the reviewer and would have liked to assess this possible relation more directly! Unfortunately, we are not confident to use the available MUS81 antibody (Higgins et al., 2008) as in our hands it also produces signal in the *mus81-2* mutant background. There is the possibility that the mutant still expresses a truncated version of MUS81 and that the antibody (that yields very reasonable signals and signal numbers) actually faithfully records MUS81-dependent recombination events. This needs to be clarified first.

With respect to the other suggestion, we indeed performed a cross between *atm-2* and *msh4* including FTL marker genes. Results confirm the involvement of class II factors in the ATM-dependent recombination increase and are shown in Figure 5.

Point 10. *I assume that the slight increase in SC width the authors also report is somehow related to this perturbation in pachytene, although clearly it doesn't appear to affect formation of both types of COs. Thus as things stand they have made an interesting observation however further analysis is required to more fully understand its implications. In fact figuring out whether it is the increased number of DSBs that increases axis length or the presence of Class II recombination intermediates would be very interesting.*

RESPONSE: We believe that ATM activity directly influences the axis/the SC (see extended discussion). Furthermore, we show that mutation of FANCM had no effect on inter-axis distances (Suppl. Figure 9C) and therefore rule out that the presence of additional class II recombination intermediates affects SC-width per se.

Point 11. *That said it would require quite an additional amount of experimentation and it is obviously not the main thrust of this current study. Nevertheless, I think that the authors could perhaps include some further discussion of there observation as it stands.*

Overall, this is very sound study, that has introduced a (for the plant field at least) a novel method to assess DSB formation. For the plant meiosis community this is a significant contribution and does clarify that in relation to the effect on DSB formation, the role of ATM more closely mirrors budding yeast rather than mouse.

RESPONSE: We thank the reviewer for the profound considerations and extended the discussion accordingly.

Adachi, S., Minamisawa, K., Okushima, Y., Inagaki, S., Yoshiyama, K., Kondou, Y., Kaminuma, E., Kawashima, M., Toyoda, T., Matsui, M., Kurihara, D., Matsunaga, S., and Umeda, M. (2011). Programmed induction of endoreduplication by DNA double-strand breaks in Arabidopsis. Proc Natl Acad Sci U S A 108, 10004-10009. Amiard, S., Depeiges, A., Allain, E., White, C.I., and Gallego, M.E. (2011). Arabidopsis ATM and ATR Kinases Prevent Propagation of Genome Damage Caused by Telomere Dysfunction. Plant Cell.

Bohmdorfer, G., Schleiffer, A., Brunmeir, R., Ferscha, S., Nizhynska, V., Kozak, J., Angelis, K.J., Kreil, D.P., and Schweizer, D. (2011). GMI1, a structural-maintenance-of-chromosomes-hinge domain-containing protein, is involved in somatic homologous recombination in Arabidopsis. Plant J 67, 420-433.

Culligan, K.M., Robertson, C.E., Foreman, J., Doerner, P., and Britt, A.B. (2006). ATR and ATM play both distinct and additive roles in response to ionizing radiation. Plant J 48, 947-961.

Friesner, J.D., Liu, B., Culligan, K., and Britt, A.B. (2005). Ionizing radiation-dependent gamma-H2AX focus formation requires ataxia telangiectasia mutated and ataxia telangiectasia mutated and Rad3-related. Mol Biol Cell 16, 25662576.

Garcia, V., Bruchet, H., Camescasse, D., Granier, F., Bouchez, D., and Tissier, A. (2003). AtATM is essential for meiosis and the somatic response to DNA damage in plants. Plant Cell 15, 119-132.

Grelon, M., Vezon, D., Gendrot, G., and Pelletier, G. (2001). AtSPO11-1 is necessary for efficient meiotic recombination in plants. EMBO J 20, 589-600.

Hartung, F., Wurz-Wildersinn, R., Fuchs, J., Schubert, I., Suer, S., and Puchta, H. (2007). The Catalytically Active Tyrosine Residues of Both SPO11-1 and SPO11-2 Are Required for Meiotic Double-Strand Break Induction in Arabidopsis. Plant Cell 19, 3090-3099.

Higgins, J.D., Buckling, E.F., Franklin, F.C., and Jones, G.H. (2008). Expression and functional analysis of AtMUS81 in Arabidopsis meiosis reveals a role in the second pathway of crossing-over. Plant J 54, 152-162.

Hisanaga, T., Ferjani, A., Horiguchi, G., Ishikawa, N., Fujikura, U., Kubo, M., Demura, T., Fukuda, H., Ishida, T., Sugimoto, K., and Tsukaya, H. (2013). The ATM-dependent DNA damage response acts as an upstream trigger for compensation in the fas1 mutation during Arabidopsis leaf development. Plant Physiol 162, 831-841.

Kurzbauer, M.T., Pradillo, M., Kerzendorfer, C., Sims, J., Ladurner, R., Oliver, C., Janisiw, M.P., Mosiolek, M., Schweizer, D., Copenhaver, G.P., and Schlogelhofer, P. (2018). Arabidopsis thaliana FANCD2 Promotes Meiotic Crossover Formation. Plant Cell 30, 415-428.

Liu, C.H., Finke, A., Diaz, M., Rozhon, W., Poppenberger, B., Baubec, T., and Pecinka, A. (2015). Repair of DNA Damage Induced by the Cytidine Analog Zebularine Requires ATR and ATM in Arabidopsis. Plant Cell.

Lorkovic, Z.J., Park, C., Goiser, M., Jiang, D., Kurzbauer, M.T., Schlogelhofer, P., and Berger, F. (2017). Compartmentalization of DNA Damage Response between Heterochromatin and Euchromatin Is Mediated by Distinct H2A Histone Variants. Curr Biol 27, 1192-1199.

Ricaud, L., Proux, C., Renou, J.P., Pichon, O., Fochesato, S., Ortet, P., and Montané, M.H. (2007). ATM-mediated transcriptional and developmental responses to gamma-rays in Arabidopsis. PLoS ONE 2, e430.

Roitinger, E., Hofer, M., Kocher, T., Pichler, P., Novatchkova, M., Yang, J., Schlogelhofer, P., and Mechtler, K. (2015). Quantitative phosphoproteomics of the ataxia telangiectasia-mutated (ATM) and ataxia telangiectasia-mutated and rad3-related (ATR) dependent DNA damage response in Arabidopsis thaliana. Mol Cell Proteomics 14, 556-571. Sims, J., Copenhaver, G.P., and Schlogelhofer, P. (2019). Meiotic DNA Repair in the Nucleolus Employs a Nonhomologous End-Joining Mechanism. Plant Cell 31, 2259-2275.

Stacey, N.J., Kuromori, T., Azumi, Y., Roberts, G., Breuer, C., Wada, T., Maxwell, A., Roberts, K., and Sugimoto-Shirasu, K. (2006). Arabidopsis SPO11-2 functions with SPO11-1 in meiotic recombination. Plant J 48, 206-216.

Su, C., Zhao, H., Zhao, Y., Ji, H., Wang, Y., Zhi, L., and Li, X. (2017). RUG3 and ATM synergistically regulate the alternative splicing of mitochondrial nad2 and the DNA damage response in Arabidopsis thaliana. Scientific reports 7, 43897.

Uanschou, C., Siwiec, T., Pedrosa-Harand, A., Kerzendorfer, C., Sanchez-Moran, E., Novatchkova, M., Akimcheva, S., Woglar, A., Klein, F., and Schlögelhofer, P. (2007). A novel plant gene essential for meiosis is related to the human CtIP and the yeast COM1/SAE2 gene. EMBO J 26, 5061-5070.

Vespa, L., Couvillion, M., Spangler, E., and Shippen, D.E. (2005). ATM and ATR make distinct contributions to chromosome end protection and the maintenance of telomeric DNA in Arabidopsis. Genes Dev 19, 2111-2115. Vespa, L., Warrington, R.T., Mokros, P., Siroky, J., and Shippen, D.E. (2007). ATM regulates the length of individual telomere tracts in Arabidopsis. Proc Natl Acad Sci U S A 104, 18145-18150.

Wang, Z., Schwacke, R., and Kunze, R. (2016). DNA Damage-Induced Transcription of Transposable Elements and Long Non-coding RNAs in Arabidopsis Is Rare and ATM-Dependent. Molecular plant 9, 1142-1155.

Waterworth, W.M., Footitt, S., Bray, C.M., Finch-Savage, W.E., and West, C.E. (2016). DNA damage checkpoint kinase ATM regulates germination and maintains genome stability in seeds. Proc Natl Acad Sci U S A.

Yoshiyama, K.O., Kobayashi, J., Ogita, N., Ueda, M., Kimura, S., Maki, H., and Umeda, M. (2013). ATM-mediated phosphorylation of SOG1 is essential for the DNA damage response in Arabidopsis. EMBO Rep 14, 817-822.

TPC2020-RA-00768D 1st Editorial decision – *request revisions* Oct. 25, 2020

We have received reviews of your manuscript entitled "ATM Controls Meiotic DNA Double-Strand Break Formation and Recombination and Affects Synaptonemal Complex Organization in Plants." Thank you for submitting your best work to The Plant Cell. The editorial board agrees that the work you describe is substantive, falls within the scope of the journal, and may become acceptable for publication, pending revision and potential re-review.

We ask you to pay attention to the following points in preparing your revision: All three reviewers have appreciated that the manuscript has been much improved. Reviewer #3 is satisfied with the current state of the manuscript,

however, reviewers 1 and 2 still have some major concerns. Reviewers #1 and 2 feel that some important controls are missing. Reviewer #1 is concerned that the new assay to detect DSBs should be validated with a proper control that is missing. Reviewer #2 insists that a WT control should be included in each independent experiment. Some of the conclusions e.g. that *atm lig4* behaves differently than *atm* seem to lack statistical support and some other conclusions should be mitigated (e.g. "genome-wide effects" of ATM mutation not supported by data or dependence of ATM effects on MUS81).

We should stress that we are reluctant to see manuscripts undergoing multiple rounds of revision and would be unlikely to offer you more than one chance to satisfy the reviewers.

A note from the Editor-in-Chief: We are trying to make a concerted effort to change green/red comparisons to green/magenta to make our figures understandable to those with color vision deficiencies. We noticed that a small number of your figures utilize red/green contrasts, so as you prepare the final version of the figures, please check the figures for red/green color use. Magenta is a good substitute for red, even if the data are labeled as "RFP" or "mCherry" - readers will understand that colors can be changed, and indeed, it can be adjusted relatively quickly by using the "hue" setting in Photoshop or similar software. For example, the following figures may need to be corrected: the shading in Figure 3B, Figures 4A, 5A, and you might want to check over the Supplemental Figures for similar comparisons. Please note that color changes do not need to be highlighted or tracked in the revised manuscript, but could be noted in the cover letter or response document.

**TPC2020-RA-00768DR1 1st Revision received**	**Dec. 18, 2020**

-- Reviewer comments (with **Authors response in bold**):

We are very grateful for the critical evaluation of our manuscript. We attentively read the editor's and reviewer's comments and prepared an accordingly revised manuscript. We have also formatted the manuscript files and figures following the instructions of *The Plant Cell*. Please find a point-by-point response to the concerns raised below (remarks are kept in *italics* and our response in regular letters). All changes made to the manuscript are highlighted in the additional manuscript file as requested.

Editor comments:

We have received reviews of your manuscript entitled "ATM Controls Meiotic DNA Double-Strand Break Formation and Recombination and Affects Synaptonemal Complex Organization in Plants." Thank you for submitting your best work to The Plant Cell. The editorial board agrees that the work you describe is substantive, falls within the scope of the journal, and may become acceptable for publication, pending revision and potential re-review.

We ask you to pay attention to the following points in preparing your revision: All three reviewers have appreciated that the manuscript has been much improved. Reviewer #3 is satisfied with the current state of the manuscript, however, reviewers 1 and 2 still have some major concerns. Reviewers #1 and 2 feel that some important controls are missing. Reviewer #1 is concerned that the new assay to detect DSBs should be validated with a proper control that is missing. Reviewer #2 insists that a WT control should be included in each independent experiment. Some of the conclusions e.g. that atm lig4 behaves differently than atm seem to lack statistical support and some other conclusions should be mitigated (e.g. "genome-wide effects" of ATM mutation not supported by data or dependence of ATM effects on MUS81).

RESPONSE: All points have been addressed. Please refer to the "reviewer section" below.

We are trying to make a concerted effort to change green/red comparisons to green/magenta to make our figures understandable to those with color vision deficiencies. We noticed that a small number of your figures utilize red/green contrasts, so as you prepare the final version of the figures, please check the figures for red/green color

use. Magenta is a good substitute for red, even if the data are labeled as "RFP" or "mCherry" - readers will understand that colors can be changed, and indeed, it can be adjusted relatively quickly by using the "hue" setting in Photoshop or similar software. For example, the following figures may need to be corrected: the shading in Figure 3B, Figures 4A, 5A, and you might want to check over the Supplemental Figures for similar comparisons. Please note that color changes do not need to be highlighted or tracked in the revised manuscript, but could be noted in the cover letter or response document.

RESPONSE: All mentioned figures have been corrected; there are no more occurrences of red and green next to each other.

Reviewer #1 (Comments for the Author):

The manuscript has been much improved, and many concerns have been addressed. However, I do have some important remaining concerns.

Point 1. I still disagree that the analyzed pictures provide a "solid base" for the statement that atm lig4 behaves differently than atm. Indeed the numbers are: 4 cells with bridges among 53 cells in atm, and 2/98 in atm lig4. 4/53 is not statistically different from 2/98! (these two observations can be sampled from a single theorical population with 4% of positive cells). These numbers need to be increased or the corresponding conclusion (bridges in atm depend on LIG4/NHEJ) should be deleted.

RESPONSE: We followed the reviewer's suggestion and removed the data concerning *atm lig4* double mutants together with the respective conclusion.

Point 2. Lack of negative controls of the novel essay to detect DSB. The authors point to the "proof of principle" experiment in S. cerevisiae, but there is no negative control in that experiment too. As this is the first description of this approach, it is crucial to have a proper negative control to prove that the detected signal reflects DSBs. I understand that providing the "SPO11-1 catalytic dead control" is difficult. However, the authors could cross their SPO11-1 tagged lines with a mutant defective in DSB formation. In such a background (e.g prd1, prd2, dfo...) no signal should be detected.

Again, this is a potentially very nice essay, that needs to be fully validated to be adopted by the community.

RESPONSE: We agree with the reviewer that a negative control for the new assay is missing in the manuscript. Unfortunately, we were not able to perform the required crosses and collect sufficient amounts of plant material to perform the suggested experiments in the given timeframe but we performed the requested proof-of-principle control in the frame of a different project concerning meiotic DNA DSB formation and repair in *Schizosaccharomyces pombe*. We are happy to share the results with you and reviewer 1 (see "Additional Figure" at the end of this document) but are hesitant to add them to the given manuscript, since they are part of another project that we expect to finish (and publish) soon. In brief, we performed the DSB quantification assay with a *S. pombe* strain exclusively expressing Rec12 (Spo11) tagged with 18xmyc, in wild type and *rec10-155* mutants. Rec10 is part of the *S. pombe* linear elements and needed to stimulate Rec12-mediated DSB formation. In *rec10-155* hypomorphic mutants, meiotic DSB formation and recombination is strongly reduced (PMID: 28469148; PMID: 21387406). The results and panels have been prepared to be in principle an additional supplementary figure, but it is at the discretion of the editor and reviewer to decide, if this figure should be included in the manuscript.

Point 3. L248. "Mutation of ATM leads to a genome-wide increase in recombination". This is an overstatement as no increase in detected on chromosomes 2 and 4 (and not in all intervals in FTLs experiments)

RESPONSE: To avoid this overstatement we removed the subtitle. No further such claim is made in the text.

Point 4. Minor: L287. It would be interesting to provide the proportion of chromatids with 0, 1, 2, 3 CO in wild type and mutant. Is the distribution different.

RESPONSE: We agree with the reviewer and included this information in SFigure 7. The occurrence of multi-CO chromosomes is indeed higher in *atm* mutants.

Reviewer #2 (Comments for the Author):

The revised manuscript has been greatly improved. I appreciate that the authors have answered most of my concerns raised during the first round of review. However, two questions are still remained.

Point 1. I don't want to argue with the authors. I do agree that wild type phenotypes including meiotic morphology have been shown many times in different papers or even in one paper. However, I do insist that wild type is a control and should be conducted in each independent experiment. For example, in SFigure 8, in addition to the A without WT (acceptable), the SFigure 8 lacks wild type and other controls such as single mutant.

RESPONSE: We followed the request of the reviewer and added images of wild-type chromatin spreads to the new Figure 1.

We would like to point out, that the seed count data for wild type and *atm-2* plants are actually not missing, but mentioned in the first chapter of the results part and displayed in (current) Figure 1 (data moved from previous SFigure 1). All counts were performed in a single experiment, with all analyzed genotypes grown side-by-side at the same time. Single mutant values for *msh4* and *mus81-2* were measured at the same time and are now also included in SFigure 8B. Additionally, we provide panels for the *msh4* and *mus81-2* single mutants.

Point 2. We know that the fertility has been affected by many factors with genetic and environment or combined each other. According to the single interval of I5d, they found that CO number in the double mutants of atm-2 mus81-2 and atm-2 msh4 is significantly increased compared with each single mutant of mus81-2 or msh4, but this does not reflect the total number of CO in the double mutants.

RESPONSE: The reviewer is correct, the measurements in the intervals represent the CO numbers (irrespective if formed via CO I or CO II mechanisms) just for this one interval. It is generally accepted that the relative changes in CO frequencies measured in this interval in different mutant backgrounds (in our case *msh4* and *mus81-2*) will allow general conclusions on factors influencing CO numbers.

Point 3. Therefore, it is hard to explain that the seeds in number is not significantly different between atm-2 mus81-2 and atm-2 msh4.

RESPONSE: We would like to point out that fertility in *atm-2 mus81-2* and *atm-2 msh4* double mutants is compromised by severe DNA repair defects (please compare to SFigure 8). Since the seed counts of *atm2 msh4* and *atm-2 mus81-2* are very low, no statistically significant difference between them could be observed.

Point 4. Also the control of atm single mutant is referred in the SFigure 1. I thought that comparison of seed number may be more difficulty, staining of pollen grain viability could be an alternative choice.

RESPONSE: We agree in principle with the reviewer, but the seed counts (and also true for potential pollen viability tests) represent a supplementary information, have been published before and do not add information to our analysis. Here we were focusing on recombination events in surviving pollen grains.

Point 5. The other question is that the authors are confident for their data set regarding the analysis of increase of COs in atm-2. Figure 5B clearly shows that the increased CO in atm should be both MUS81-dependent and -independent. That authors also realized this and provided an speculation in line 331-334 on page 13 "We therefore conclude that not only MUS81, but also further class II CO mediators like FANCD2 (Kurzbauer et al., 2018) are responsible for the elevated recombination rates in atm-2 mutants, while class I CO factors are not involved". In my opinion, it would be more reasonable to draw the conclusion that the increased CO in atm is at least partially MUS81-dependnet.

RESPONSE: We agree with the reviewer and believe that their conclusion is identical to ours.

Reviewer #3 (Comments for the Author):

This substantially revised manuscript by Kurzbauer et al presents their characterization of an Arabidopsis mutant that is defective in the DNA damage sensing ATM kinase. The authors present evidence that indicates that ATM is important for limiting the number of meiotic DNA double-strand breaks (DSBs) together with limiting the number of interference insensitive genetic crossovers. They present a novel method to determine the level of DSBs. Additionally they provide evidence that loss of the protein affects organization of the chromosome axis and synaptonemal complex (SC).
I reviewed (Reviewer 3) the earlier version of the manuscript raising a number of points/queries. The authors have addressed all of these satisfactorily. I note that they have also addressed the points made by the other two reviewers. One comment I would make to a point raised by reviewer 1 (point 5) in relation to excluding chromosomes 2 and 4 from the analysis of CO frequency (Lines 282-292). The authors are correct in their assertion that these chromosomes can behave atypically relative to chromosomes 1, 3 and 5. This is very probably due to the NORs which do seem to influence behaviour, likely early alignment. For example, in the asynaptic mutant asy1 which exhibits highly reduced CO formation, the residual COs are disproportionately found on the NOR chromosomes. Hence, a difference in behaviour between the two sets of chromosomes in the atm mutant may not be so unusual.

Overall the authors have gone to considerable lengths to respond to the previous comments. They have significantly strengthened the manuscript and as such to it is a valuable contribution to the plant meiosis field.

RESPONSE: We are glad to hear that reviewer #3 is satisfied with the revised manuscript. Many thanks!

We would like to thank all three reviewers for assessment of our work and for their sincere engagement and the time they invested. Their critical and constructive remarks considerably helped to improve the quality of the experiments and the manuscript.

TPC2020-RA-00768DR1 2nd Editorial decision – *accept pending minor revisions* Dec. 23, 2020

We have received reviews of your manuscript entitled "ATM Controls Meiotic DNA Double-Strand Break Formation and Recombination and Affects Synaptonemal Complex Organization in Plants." On the basis of the advice received, the board of reviewing editors would like to accept your manuscript for publication in The Plant Cell. This acceptance is contingent on revision based on the comments of our reviewers. In particular, please consider the following:

We felt that you have addressed the main concerns of the referees and that your work represents a significant advance to the field of meiosis in plants, in particular with regards the function of ATM in limiting the number of meiotic DSBs and in the organization of the chromosome axis and synaptonemal complex. In addition, you describe a new method for measuring the number of DSBs in the cell that might become a useful assay for the community. The revised manuscript is thus acceptable for publication. However, it should be noted that the additional supplementary figure showing validation of the DSB-quantification method in *Schizosaccharomyces pombe* should be included in the final version. In its absence, the novelty of the manuscript would be diminished and the current description of the assay, lacking a negative control, would not stand alone.

TPC2020-RA-00768DR2 2nd Revision received Jan. 19, 2021

Changes to the manuscript due to incorporation of SIM-TIRF experiments in *S. pombe*:
+Manuscript: The Results part now mentions the *S. pombe* experiment on page 7.

+Manuscript: The Materials & Methods part was extended and now contains detailed information on the experimental procedures performed in *S. pombe*.
+Supplemental Figure 4 now also contains results from *S.pombe*. The figure and legend were modified accordingly.
+Supplemental Tables 1 and 4 were extended and now include *S.pombe* results.
+Supplemental Dataset 2 was extended to contain information on the statistical anayliss performed on SIM-TIRF experiments in *S. pombe.*

TPC2020-RA-00768DR2 3rd Editorial Decision – *accept* **Jan. 19, 2021**

We are pleased to inform you that your paper entitled "ATM Controls Meiotic DNA Double-Strand Break Formation and Recombination and Affects Synaptonemal Complex Organization in Plants" has been accepted for publication in The Plant Cell, pending a final minor editorial review by journal staff. At this stage, your manuscript will be evaluated by a Science Editor with respect to its presentation of scientific content, compliance with journal policies, and presentation for a broad readership

Final acceptance from Science Editor **Jan. 29, 2021**
